# Clinical, polysomnographic, and psychological profiles across questionnaire-defined symptom groups in obstructive sleep apnea

**DOI:** 10.3389/fmed.2026.1948049

**Published:** 2026-09-30

**Authors:** Jiahui Xu, Yufei Tian, Wei Luo, Ziyang Zhai, Xiaoling Wang, Tiantian Wang, Qinglin Xu, Xingyue Hu, Lisan Zhang

**Affiliations:** 1 Department of Neurology/Center for Sleep Medicine, Sir Run Run Shaw Hospital, School of Medicine, Zhejiang University, Hangzhou, China; 2 College of Biomedical Engineering & Instrument Science, Zhejiang University, Hangzhou, China; 3 Department of Pharmacy, Xiasha Campus, Sir Run Run Shaw Hospital, School of Medicine, Zhejiang University, Hangzhou, China

**Keywords:** excessive daytime sleepiness, obstructive sleep apnea, polysomnography, sleep quality, symptom groups

## Abstract

Obstructive sleep apnea (OSA) has heterogeneous symptom presentations that are not fully captured by the apnea–hypopnea index (AHI). We aimed to identify demographic, polysomnographic, and psychological profiles across four questionnaire-defined symptom groups in OSA patients. This retrospective cross-sectional study included 789 treatment-naïve patients with OSA (AHI ≥5 events/h) who underwent overnight polysomnography and neuropsychological assessment between May 2021 and August 2025. Participants were classified into four symptom-groups: low symptom burden, poor sleep quality only, excessive daytime sleepiness (EDS) only, and combined poor sleep quality and EDS, using the Pittsburgh Sleep Quality Index and the Epworth Sleepiness Scale. Multinomial logistic regression was used to identify factors associated with these symptom-groups, with the low symptom burden group as the reference. Results showed that the poor sleep quality only symptom-group was most prevalent (49.0%), followed by the low symptom burden group (29.8%), the combined group (14.3%), and the EDS-only group (6.8%). Female sex, lower BMI, altered sleep continuity and sleep-stage distribution were associated with the poor sleep quality only group, whereas younger age, shorter sleep onset latency and a shorter maximum hypopnea duration were associated with the EDS-only group. Higher PHQ-9 and CFS scores were consistently associated with all three higher symptom-burden groups (all *P* < 0.001), with the largest effect estimates observed for the combined group. Higher GAD-7 scores were also associated with the poor sleep quality only and combined groups, whereas the association between GAD-7 score and the EDS-only group was less precise and only nominally significant. Several additional sleep-stage, stage- or position-specific respiratory, and limb-movement associations were nominal and should be considered hypothesis-generating. Several conventional hypoxemia measures, including TST90, mean oxygen desaturation, and mean nocturnal oxygen saturation, were not significantly associated with any symptom-group in the primary adjusted analyses. The overall pattern was generally similar in the sensitivity analyses, although several nominal associations were attenuated. Overall, questionnaire-defined OSA symptom groups differed in demographic, polysomnographic, and psychological characteristics that were not fully captured by AHI alone, supporting multidimensional clinical assessment while considering the need for independent validation.

## 1 Introduction

Obstructive sleep apnea (OSA) is a prevalent sleep-related breathing disorder characterized by recurrent upper airway collapse, leading to intermittent hypoxia and sleep fragmentation. The condition affects approximately one billion adults worldwide (1) and is independently associated with cardiovascular morbidity (2–6), metabolic dysregulation (7, 8), and neurocognitive decline (9). Traditionally, OSA severity is classified using the apnea-hypopnea index (AHI). However, the AHI shows a limited correlation with symptom burden; patients with comparable AHI values frequently exhibit markedly different clinical presentations, ranging from excessive daytime sleepiness (EDS) (10) to sleep-maintenance insomnia (11) or even asymptomatic disease (12). This discordance limits the clinical utility of AHI-centered management strategies and highlights the need for more multidimensional, symptom-informed approaches.

To address this heterogeneity, seminal work using the Icelandic Sleep Apnea Cohort applied latent class analysis to stratify patients according to symptom profiles (12). This landmark study identified three distinct phenotypes: “excessively sleepy”, “disturbed sleep”, and “minimally symptomatic”. These clusters were subsequently validated and expanded across diverse international populations and cohorts (13–20). These phenotypes have demonstrated divergent trajectories in responsiveness to continuous positive airway pressure (CPAP) therapy (17, 21, 22) and in long-term cardiovascular prognosis (16–19). Nevertheless, despite recognition of these clinically meaningful clusters, employing cluster analysis for symptomatic classification of OSA typically generates data-driven subtypes that are difficult to replicate and implement in routine clinical practice. In addition, the factors associated with insomnia-predominant versus sleepiness-predominant presentations remain incompletely understood. Although univariate analyses suggest that the sleepy subtype tends to be younger and more obese (16, 19, 23), findings across studies have been inconsistent (13–15, 20, 21). Furthermore, the independent contributions of detailed polysomnographic parameters, adjusted for potential confounding by age, sex, and comorbidities, have not been rigorously evaluated.

Building on previous cluster-based studies, we used a rule-based classification that cross-classified two questionnaire dimensions: subjective sleep quality assessed using the Pittsburgh Sleep Quality Index (PSQI) and excessive daytime sleepiness assessed using the Epworth Sleepiness Scale (ESS). This approach was intended as a pragmatic operationalization of clinically relevant symptom combinations rather than as a new phenotype system or an alternative shown to be superior to latent class or cluster analysis. It permits transparent group assignment without cohort-specific model fitting, whereas data-driven methods can capture broader latent symptom structures. We therefore compared demographic, polysomnographic, and psychological characteristics across the four questionnaire-defined symptom groups.

## 2 Methods

### 2.1 Study design and population

We conducted a retrospective cross-sectional study of consecutive patients who underwent overnight polysomnography at the Sleep Medicine Center of Sir Run Run Shaw Hospital, Zhejiang University School of Medicine, between May 2021 and August 2025. Eligible participants were patients aged ≥ 16 years with treatment-naïve obstructive sleep apnea (OSA), defined as an apnea-hypopnea index (AHI) ≥ 5 events/h. Exclusion criteria were: (i) prior or ongoing OSA-specific treatment, including continuous positive airway pressure (CPAP), oral appliances, or upper airway surgery; (ii) missing PSQI and/or ESS data required for symptom classification; (iii) central sleep apnea (central apnea index > 5 events/h or central events accounting for > 50% of all respiratory events). A total of 1,671 patients were screened. Of these, 71 were aged <16 years and 370 had an AHI <5 events/h, leaving 1,230 patients with OSA who met the age and AHI criteria. We then excluded 13 patients with central sleep apnea, 29 with prior or ongoing OSA-specific treatment, and 399 with missing PSQI and/or ESS data required for symptom-group classification. The final analysis therefore included 789 treatment-naïve patients with OSA (Figure 1). The study was approved by the Ethics Committee of Sir Run Run Shaw Hospital and conducted in accordance with the Declaration of Helsinki. The requirement for informed consent was waived because of the retrospective study design.

**Figure 1:**
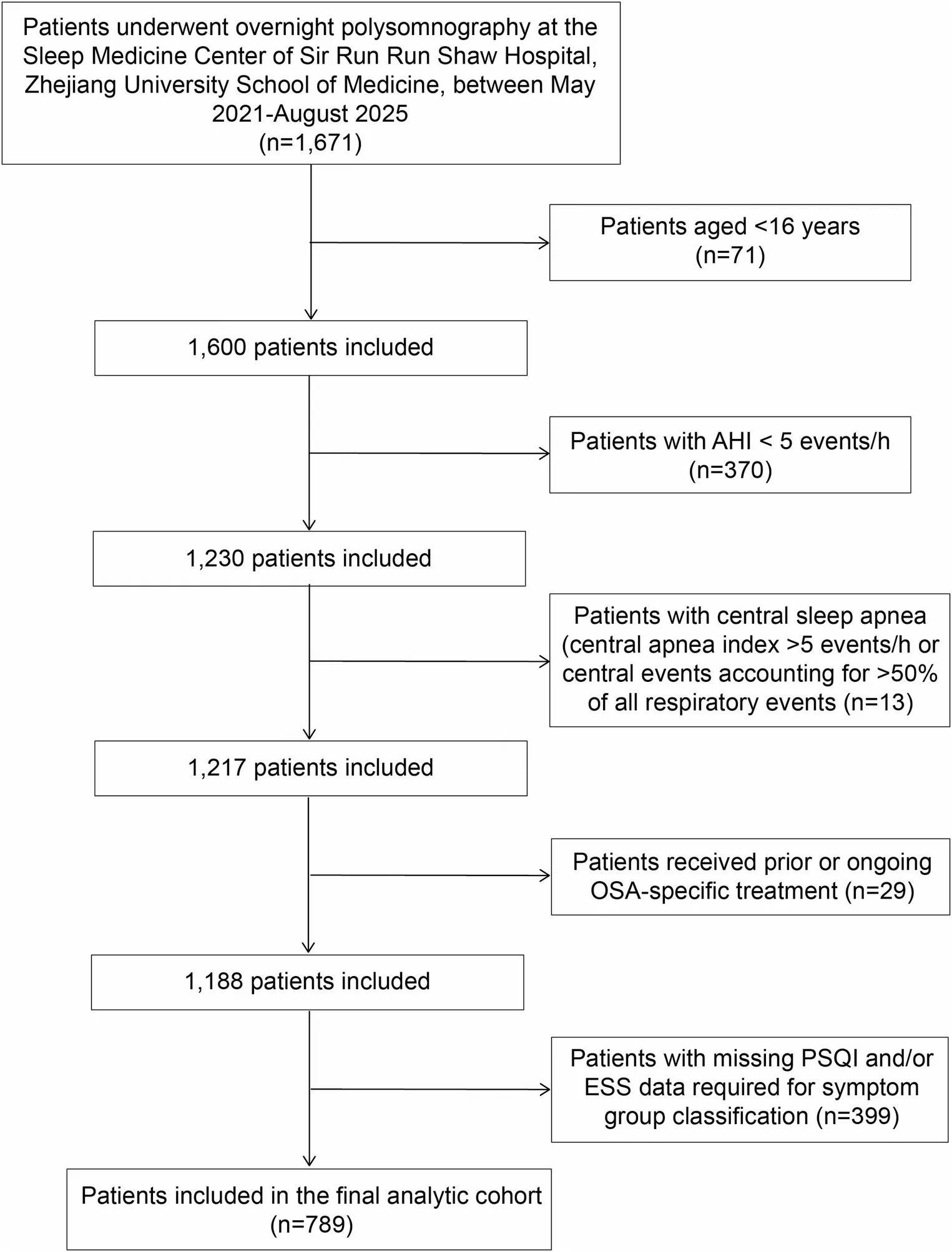
Flow diagram of participant screening and selection.

### 2.2 Clinical assessments and symptom-group definitions

Demographic characteristics and anthropometric data were extracted from clinical records and assessments. Body mass index (BMI) was calculated as body weight in kilograms divided by height in meters squared (kg/m^2^). A binary indicator of any coexisting sleep disorder was defined as the presence of at least one documented diagnosis of restless legs syndrome, REM sleep behavior disorder, or narcolepsy. Participants with more than one coexisting sleep disorder were counted only once in this category. Medical comorbidities were extracted from the clinical records and coded as separate binary variables. Hypertension, diabetes, and cardiovascular disease were not mutually exclusive; therefore, an individual participant could be included in more than one comorbidity category. Cardiovascular disease included myocardial infarction, coronary artery disease, congestive heart failure, and stroke.

Before polysomnography, all participants were asked to complete a standardized battery of validated Chinese-language self-report questionnaires. Subjective sleep quality was assessed using the Pittsburgh Sleep Quality Index (PSQI) (24), and excessive daytime sleepiness (EDS) was assessed using the simplified Chinese version of the Epworth Sleepiness Scale (ESS) (25). Anxiety and depressive symptoms were assessed using validated Chinese versions of the Generalized Anxiety Disorder-7 (GAD-7) (26) and Patient Health Questionnaire-9 (PHQ-9) (27), respectively. Fatigue severity was assessed using the validated Chinese version of the Chalder Fatigue Scale (CFS) (28).

Participants were classified into four questionnaire-defined symptom groups by cross-classifying the PSQI and ESS scores. Poor sleep quality was defined as a PSQI score > 7, a cutoff supported by validation of the Chinese version of the PSQI (24). EDS was operationally defined as an ESS score ≥ 10; the simplified Chinese version of the ESS has demonstrated satisfactory reliability and validity in Chinese patients evaluated for OSA (25). The four groups were: (i) low symptom burden: PSQI ≤ 7 and ESS < 10; (ii) poor sleep quality only: PSQI > 7 and ESS < 10; (iii) EDS only: PSQI ≤ 7 and ESS ≥ 10; and (iv) combined poor sleep quality and EDS: PSQI > 7 and ESS ≥ 10. These labels describe symptom patterns only across the two questionnaire dimensions used for classification. Accordingly, “poor sleep quality” does not represent a formal diagnosis of insomnia, “low symptom burden” does not indicate the absence of all OSA-related symptoms, and “only” indicates that one, rather than both, of the two questionnaire thresholds was met. This rule-based classification was used to compare combinations of the two questionnaire dimensions and was not intended to identify latent or biologically distinct OSA subtypes.

### 2.3 Polysomnography

Standard overnight Type I polysomnography was performed using a digital monitoring system (Compumedics Grael system, Australia). Recorded physiological parameters included electroencephalography, electrooculography, submental and bilateral anterior tibialis electromyography, electrocardiography, nasal airflow measured by both pressure transducer and thermistor, thoracoabdominal respiratory effort, and pulse oximetry. Sleep stages, respiratory events, electroencephalographic arousals, and limb movements were scored manually by certified sleep technologists in accordance with the American Academy of Sleep Medicine Manual for the Scoring of Sleep and Associated Events. Polysomnographic examinations performed before January 2024 were scored according to version 2.6, whereas those performed from that date onward were scored according to version 3.0. Although the referenced edition of the manual changed, the operational criteria used in our laboratory for sleep staging, respiratory-event scoring, and limb-movement scoring relevant to the present analyses were applied consistently throughout the study period. In particular, the same hypopnea criterion was used throughout: a reduction in airflow of ≥ 30% from baseline for ≥10 s, accompanied by either a ≥ 3% oxygen desaturation or an electroencephalographic arousal. The apnea-hypopnea index was calculated as the number of apneas and hypopneas per hour of total sleep time. TST90 was defined as the percentage of total sleep time spent with oxygen saturation below 90% and was calculated as the cumulative time with SpO_2_ < 90% divided by total sleep time and multiplied by 100.

### 2.4 Statistical analysis

Descriptive analyses were based on the available observations for each variable. Continuous variables were summarized as mean ± standard deviation (SD) when approximately normally distributed or median (25th, 75th percentiles) when non-normally distributed. Categorical variables were presented as *n* (%) when no missing observations, and as n/N (%) when missing observations were present, where N denoted the number of participants with available data within corresponding symptom-group. No missing-value imputation was performed. Differences among the four symptom-groups were evaluated using one-way analysis of variance or the Kruskal–Wallis test for continuous variables. Categorical variables were compared using the chi-square test or Fisher's exact test, as appropriate. Because PSQI and ESS were used to define the symptom-groups, their between-group differences were not interpreted inferentially. The score distributions were summarized descriptively, and the corresponding *P* values were not reported.

Multinomial logistic regression was performed with the low symptom burden group as the reference group. Candidate variables for the multinomial logistic regression analyses were selected using a combined clinically informed and data-informed approach. Age, sex, and BMI were retained as core demographic covariates irrespective of their statistical significance in the descriptive comparisons. Psychological factors and polysomnographic parameters were considered for subsequent regression analyses if they had established clinical relevance to OSA symptom presentation or disease severity and/or showed significant differences across symptom-groups. Two sets of models were fitted. First, a demographic model including age, sex, and body mass index (BMI) simultaneously was used to examine mutually adjusted demographic correlates of symptom-groups. Second, each polysomnographic parameter, as well as each psychological scale score, including GAD-7, PHQ-9, and CFS scores, was entered separately into a multinomial logistic regression model adjusting for age, sex, and BMI. This modeling strategy was used to avoid overfitting and unstable estimates caused by collinearity among physiologically related polysomnographic variables. Each multinomial logistic regression model was fitted using model-specific complete cases, defined as participants with non-missing data for the symptom-group outcome, the predictor of interest, and all covariates included in that model. Model convergence was assessed for each fitted multinomial logistic regression model. Coefficient estimates, standard errors, and 95% CIs were examined for evidence of estimation instability or sparse-data effects, with particular attention to the EDS-only group. The consistency of the direction and magnitude of the associations across the primary and sensitivity analyses was also considered when interpreting model stability.

All continuous predictors in the regression models were standardized using Z-score transformation before analysis, so that odds ratios could be interpreted as the change in odds per one SD increase in the predictor and compared across variables measured on different scales. To assess the robustness of the findings in clinically significant OSA, descriptive and regression analyses were repeated in patients with moderate-to-severe OSA, defined as an AHI ≥15 events/h. To evaluate the potential influence of coexisting sleep disorders, we performed an additional sensitivity analysis restricted to participants with available coexisting-sleep-disorder status and no documented diagnosis of restless legs syndrome, REM sleep behavior disorder, or narcolepsy. All multinomial logistic regression analyses were repeated using the same model specifications as in the primary analysis.

Moreover, to assess whether the associations between polysomnographic variables and symptom-group membership were sensitive to overall OSA severity, we performed additional sensitivity analyses of polysomnographic correlates with further adjustment for AHI. AHI was not evaluated as a focal predictor in these models because it served as the additional adjustment variable. For stage- and position-specific AHI and apnea or hypopnea indices, the estimates were interpreted conditionally as differences in respiratory-event distribution or composition at a comparable overall event burden. Variance inflation factors were examined to assess collinearity between the focal polysomnographic variable and AHI. Given the exploratory nature of the analyses and multiple candidate polysomnographic and questionnaire variables examined, *P* values were not adjusted for multiple comparisons. Statistical evidence was evaluated using effect estimates, 95% confidence intervals (CIs), the magnitude of *P* values, and consistency across primary and sensitivity analyses. A two-sided *P* value < 0.05 was considered nominally statistically significant. Associations supported only by *P* values close to 0.05, CIs close to the null, or inconsistent sensitivity analysis results were regarded as hypothesis-generating rather than confirmatory. All statistical analyses were performed using Python 3.9.

## 3 Results

### 3.1 Prevalence and distribution of symptom-groups

A total of 789 treatment-naïve patients with confirmed OSA and complete PSQI and ESS data required for the symptom-group classification were included in the primary analysis. Based on the predefined PSQI and ESS thresholds, poor sleep quality only was the most common symptom-group, accounting for 49.0% of the cohort (*n* = 387), followed by the low symptom burden group (29.8%, *n* = 235), the combined group (14.3%, *n* = 113), and the EDS-only group (6.8%, *n* = 54).

### 3.2 Demographic and clinical characteristics

Demographic and anthropometric characteristics differed across the four symptom-groups (Table 1). Patients with EDS only were the youngest and had the highest BMI, with a median age of 41.5 years and a median BMI of 26.5 kg/m^2^, respectively. In contrast, patients with poor sleep quality only were older and had the lowest BMI, with median values of 54.0 years and 24.1 kg/m^2^, respectively (both *P* < 0.001). Across age categories, the proportion of poor sleep quality only increased progressively, while EDS only became less frequent with increasing age (Figure 2). Because PSQI and ESS were used to define the symptom-groups, their distributions are presented descriptively in Table 1 without corresponding between-group *P* values. An opposite pattern was observed across BMI strata. The proportion of poor sleep quality only decreased as BMI increased, whereas EDS only and the combined symptom-group became more common among participants with higher BMI levels (Figure 3).

**Table 1:** General characteristics of the four symptom-groups.

Characteristics	Symptom-group				*P*
	Low symptom burden	Poor sleep quality only	EDS only	Combined	
Number	235	387	54	113	-
Sex, male, *n* (%)	188 (80.0%)	242 (62.5%)	42 (77.8%)	86 (76.1%)	**<0**.**001**
Age (years)	51.00 (37.00, 62.50)	54.00 (44.00, 62.50)	41.50 (26.3, 57.75)	47.00 (34.00, 57.00)	**<0**.**001**
BMI (kg/m^2^)	25.20 (23.39, 28.00)	24.10 (22.00, 26.30)	26.54 (24.20, 29.77)	25.30 (23.30, 29.00)	**<0**.**001**
PSQI	5.00 (3.00, 6.00)	12.00 (10.00, 15.00)	6.00 (4.00, 7.00)	11.00 (9.00, 13.00)	**-**
ESS	4.00 (2.00, 6.00)	3.00 (1.00, 6.00)	12.00 (11.00, 16.00)	13.00 (11.00, 15.00)	**-**
PHQ-9	2.00 (0.00, 5.00)	6.00 (3.00, 9.00)	6.00 (2.00, 10.00)	8.00 (4.00, 12.00)	**<0**.**001**
GAD-7	2.00 (0.00, 4.00)	4.00 (2.00, 8.00)	3.00 (0.00, 6.00)	6.00 (3.00, 9.50)	**<0**.**001**
CFS	5.00 (3.00, 7.00)	7.00 (5.00, 9.75)	8.00 (6.00, 10.00)	9.00 (7.00, 11.00)	**<0**.**001**
Any coexisting sleep disorder, n/N (%)^a^	46/207 (22.2%)	86/350 (24.6%)	15/49 (30.6%)	27/106 (25.5%)	0.653
Hypertension, *n* (%)	95 (40.4%)	147 (38.0%)	17 (31.5%)	36 (31.9%)	0.352
Diabetes, *n* (%)	24 (10.2%)	49 (12.7%)	4 (7.4%)	11 (9.7%)	0.553
Cardiovascular disease, *n* (%)^b^	38 (16.2%)	35 (9.0%)	5 (9.3%)	10 (8.8%)	**0**.**037**

Continuous variables are presented as median (25th, 75th percentiles). Categorical variables with missing observations are presented as n/N (%), where N represents the number of participants with available data. Unless otherwise indicated, percentages were calculated using the total number of participants in each symptom-group.

EDS, excessive daytime sleepiness; BMI, body mass index; PSQI, Pittsburgh Sleep Quality Index; ESS, Epworth Sleepiness Scale; PHQ-9, Patient Health Questionnaire-9; GAD-7, Generalized Anxiety Disorder-7; CFS, chalder fatigue scale.

Statistically significant results (*P* < 0.05) are shown in **bold.**

a Coexisting sleep disorders included restless legs syndrome, REM sleep behavior disorder, and narcolepsy.

b Cardiovascular disease (including myocardial infarction, coronary artery disease, congestive heart failure, and stroke).

PSQI and ESS were used to define the four symptom groups. Their distributions are therefore presented for descriptive purposes only, and the corresponding between-group *P* values are not reported.

**Figure 2:**
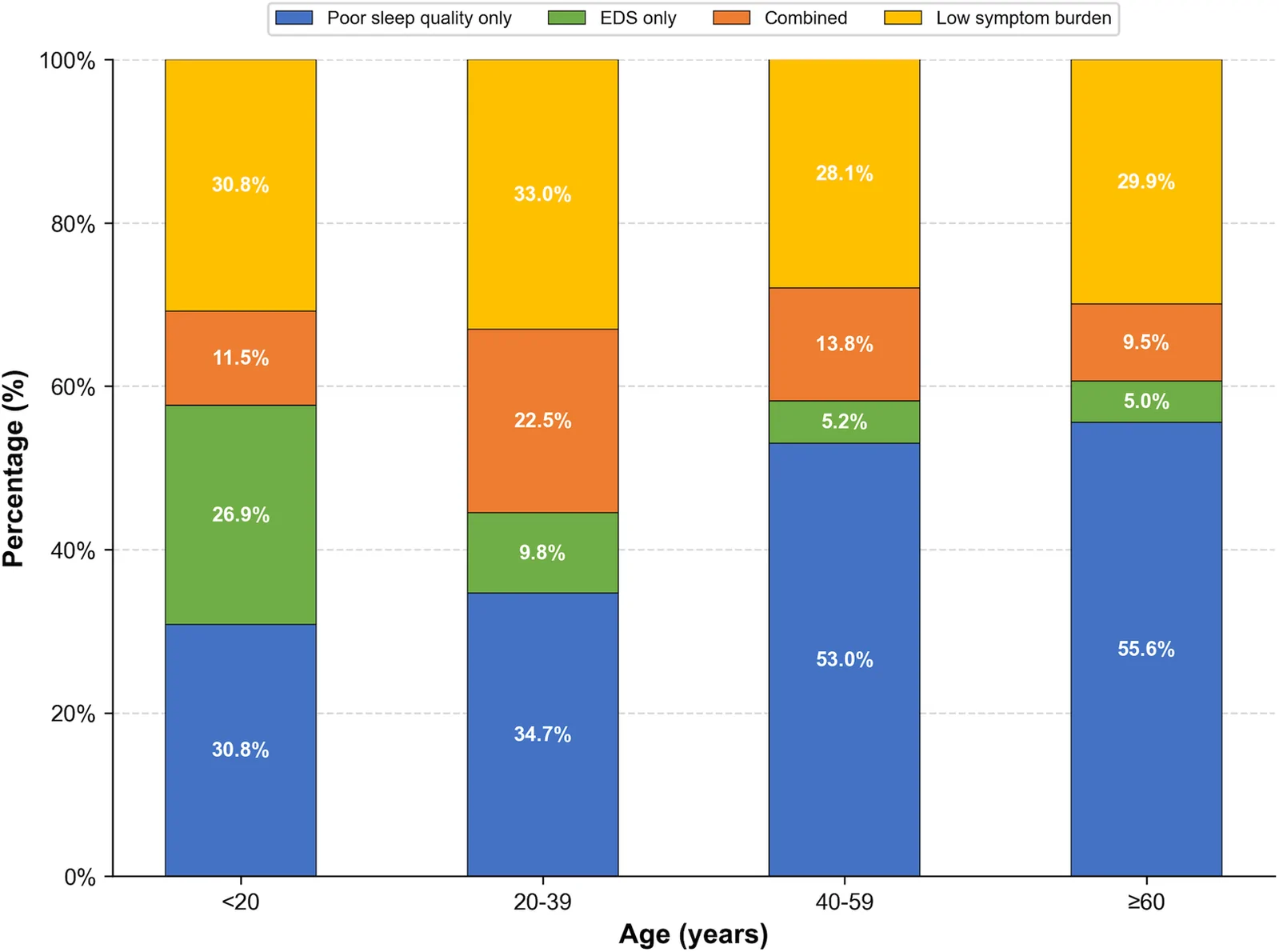
Age-stratified distribution of the four OSA symptom-groups. The proportion of poor sleep quality only (blue) rose steadily with advancing age, whereas the proportion of EDS only (green) progressively declined.

**Figure 3:**
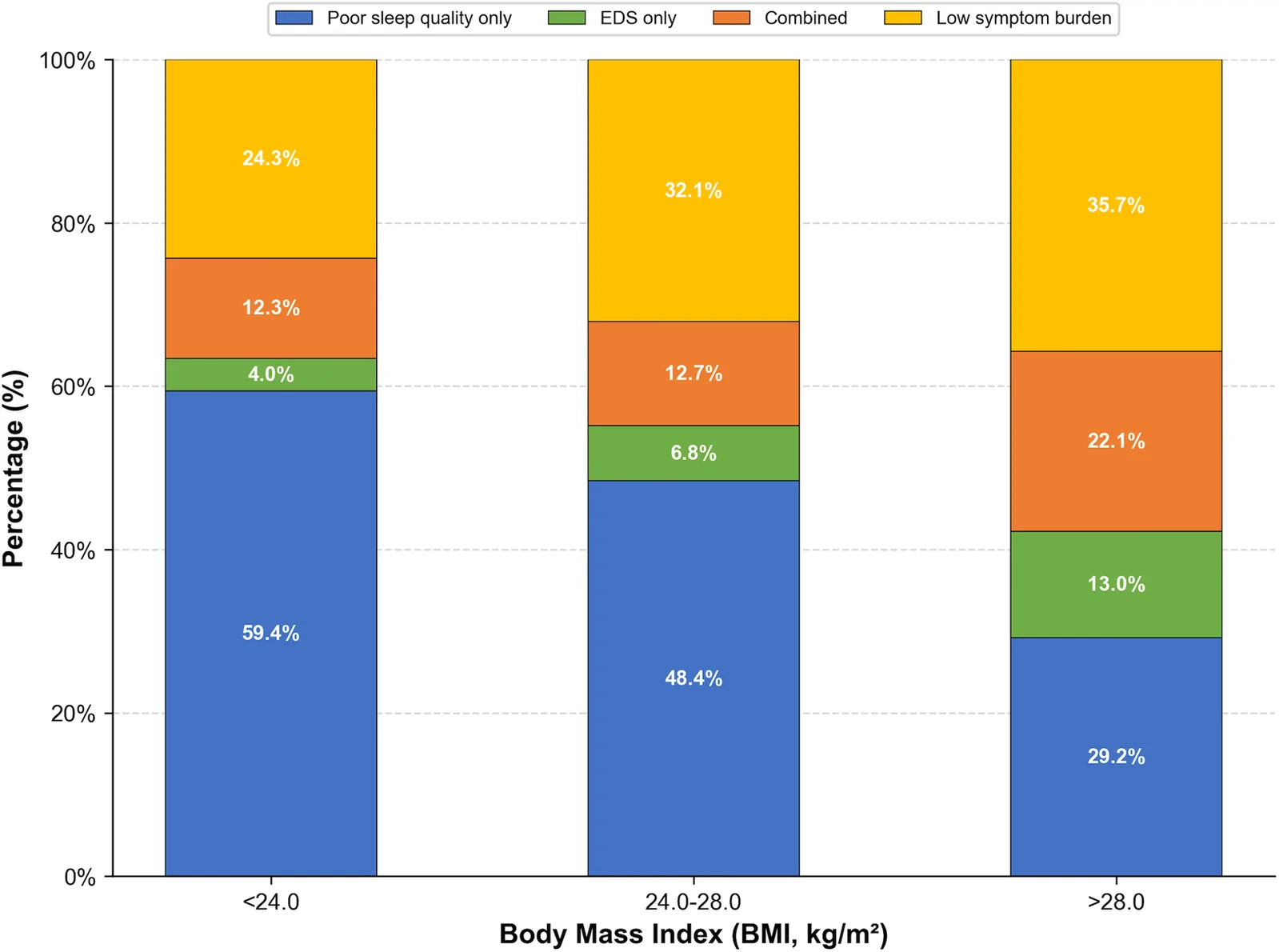
BMI-stratified distribution of the four OSA symptom-groups. The proportion of poor sleep quality only (blue) decreased as the BMI increased, whereas the proportion of EDS only (green) expanded progressively from the normal-weight to the obese category.

Psychological and fatigue-related symptom burden also differed across symptom-groups (Table 1). PHQ-9, GAD-7, and CFS scores were higher across the other three symptom groups than in the low symptom burden group. The combined symptom-group had the highest overall psychological burden (all *P* < 0.001). The prevalence of cardiovascular disease differed significantly across the four symptom groups (*P* = 0.037), whereas no significant differences were observed for hypertension and diabetes (both *P* > 0.05). The prevalence of coexisting sleep disorders was also similar across the four groups (*P* > 0.05).

### 3.3 Polysomnographic profiles

Polysomnographic characteristics differed across the four symptom-groups (Table 2). The poor sleep quality only symptom-group showed poorer sleep continuity, with the lowest sleep efficiency (median value of 75.5%), compared with 78.3% in the low symptom burden group, 81.6% in EDS only, and 78.9% in the combined symptom-group (*P* < 0.001). Sleep latency was longest in poor sleep quality only, with a median of 12.0 min, compared with 8.5 min, 4.5 min, and 7.0 min in the low symptom burden, EDS only, and combined symptom-groups, respectively (*P* < 0.001). Sleep-stage distribution and arousal indices also differed among symptom-groups. REM sleep percentage was highest in EDS only, with a median of 16.95%, compared with 14.80% in the low symptom burden group, and 15.60% in both poor sleep quality only and the combined symptom-group (*P* = 0.030). The respiration-related arousal index was lower in poor sleep quality only than in the low symptom burden and EDS only symptom-groups (*P* < 0.001), whereas spontaneous arousal index was numerically higher in poor sleep quality only than in EDS only (*P* = 0.044).

**Table 2:** Polysomnographic parameters in the four symptom-groups.

Characteristics	Symptom-group				*P*
	Low symptom burden	Poor sleep quality only	EDS only	Combined	
Number	235	387	54	113	**-**
1. Sleep stages and arousals					
Total sleep time (min)	416.50 (351.00, 471.75)	394.50 (340.25, 453.50)	438.50 (388.62, 477.50)	416.50 (368.00, 459.00)	< 0.001
Sleep efficiency (%)	78.30 (67.50, 84.90)	75.50 (64.75, 83.75)	81.55 (74.17, 90.65)	78.90 (70.00, 87.80)	< 0.001
Sleep onset latency (min)	8.50 (5.50, 16.50)	12.00 (6.50, 22.75)	4.50 1.50, 8.00)	7.00 (3.50, 16.50)	< 0.001
REM sleep latency (min)	98.00 (73.00, 155.50)	102.00 (73.25, 162.25)	84.00 (52.62, 116.88)	98.00 (59.50, 144.00)	0.004
N1% (%)	30.30 (20.50, 45.30)	25.70 (18.75, 35.35)	27.90 (18.62, 40.55)	27.90 (18.00, 40.20)	0.002
N2% (%)	36.70 (30.15, 42.95)	39.90 (33.60, 47.75)	37.30 (31.00, 46.60)	38.10 (29.40, 44.70)	0.001
N3% (%)	13.10 (6.65, 19.25)	14.70 (8.30, 22.20)	13.25 (4.03, 18.45)	13.20 (6.90, 20.10)	0.045
REM% (%)	14.80 (10.90, 18.45)	15.60 (11.30, 19.35)	16.95 (14.05, 19.38)	15.60 (12.70, 20.50)	0.030
WASO (min)	106.00 (69.25, 154.25)	109.50 (65.00, 169.75)	85.50 (46.25, 139.00)	96.50 (59.50, 149.00)	0.062
Index of sleep stage transformation (events/h)	38.84 (30.30, 53.83)	38.25 (29.45, 46.22)	35.30 (23.61, 45.45)	38.01 (28.57, 47.19)	0.215
Index of sleep to wake transitions (events/h)	5.83 (4.12, 7.77)	5.41 (3.87, 7.23)	4.85 (3.00, 6.71)	5.84 (3.69, 8.19)	0.334
Total arousal index (events/h)	31.80 (22.00, 47.50)	27.30 (19.50, 37.70)	28.25 (19.68, 42.45)	29.00 (20.90, 41.90)	0.009
Respiration-related arousal index (events/h)	17.80 (9.35, 34.10)	12.90 (6.75, 22.30)	16.95 (7.35, 33.80)	13.90 (7.90, 28.30)	< 0.001
Respiration-related arousal index: NREM (events/h)	17.30 (9.60, 35.30)	12.40 (6.50, 23.45)	16.50 (6.33, 34.90)	14.00 (7.80, 30.00)	< 0.001
Respiration-related arousal index: REM (events/h)	16.60 (7.00, 31.70)	12.50 (5.50, 24.35)	13.50 (6.40, 36.30)	14.70 (7.10, 24.70)	0.014
LM-related arousal index (events/h)	0.30 (0.10, 0.60)	0.30 (0.00, 0.60)	0.40 (0.12, 0.67)	0.40 (0.10, 0.90)	0.044
LM-related arousal index: NREM (events/h)	0.30 (0.15, 0.70)	0.30 (0.00, 0.60)	0.40 (0.12, 0.60)	0.40 (0.20, 0.90)	0.058
LM-related arousal index: REM (events/h)	0.00 (0.00, 0.00)	0.00 (0.00, 0.00)	0.00 (0.00, 0.80)	0.00 (0.00, 0.60)	0.006
PLM-related arousal index (events/h)	0.20 (0.00, 0.70)	0.00 (0.00, 0.70)	0.10 (0.00, 0.95)	0.20 (0.00, 1.20)	0.150
PLM-related arousal index: NREM (events/h)	0.20 (0.00, 0.75)	0.00 (0.00, 0.80)	0.00 (0.00, 1.05)	0.20 (0.00, 1.10)	0.168
PLM-related arousal index: REM (events/h)	0.00 (0.00, 0.00)	0.00 (0.00, 0.00)	0.00 (0.00, 0.00)	0.00 (0.00, 0.00)	0.167
Spontaneous arousal index (events/h)	10.10 (7.70, 14.35)	10.70 (7.80, 15.40)	8.85 (6.72, 12.75)	10.30 (6.80, 13.20)	0.044
Spontaneous arousal index: NREM (events/h)	10.80 (7.85, 15.35)	11.40 (8.25, 16.50)	8.90 (7.03, 13.90)	10.40 (6.90, 13.50)	0.047
Spontaneous arousal index: REM (events/h)	6.70 (3.60, 10.50)	7.30 (4.55, 11.40)	7.25 (4.03, 9.47)	7.30 (4.80, 10.80)	0.298
2. Parameters of OSA severity and hypoxemia					
AHI (events/h)	27.35 (14.99, 47.80)	18.50 (11.19, 33.61)	28.43 (12.01, 44.47)	24.74 (12.79, 43.67)	< 0.001
ODI (events/h)	15.90 (4.90, 36.50)	9.50 (3.30, 25.10)	18.45 (3.50, 37.22)	18.20 (4.00, 37.20)	< 0.001
AI (events/h)	4.54 (0.62, 17.41)	2.02 (0.33, 8.32)	3.33 (0.41, 31.24)	4.01 (0.63, 17.53)	0.001
HI (events/h)	16.64 (9.92, 25.30)	13.68 (8.07, 21.19)	13.90 (7.83, 20.78)	14.72 (9.08, 20.47)	0.014
AHI_NREM_ (events/h)	21.54 (8.29, 43.69)	14.58 (6.75, 29.67)	22.84 (6.07, 43.73)	19.17 (9.59, 39.53)	0.001
AHI_REM_ (events/h)	22.50 (10.70, 38.70)	21.50 (9.55, 35.80)	24.35 (10.50, 55.20)	25.80 (12.50, 42.50)	0.060
AHI_nonsupine_ (events/h)	25.60 (6.75, 63.05)	16.60 (5.25, 44.40)	51.85 (12.30, 82.15)	25.40 (9.90, 67.10)	< 0.001
AHI_supine_ (events/h)	33.00\ (19.95, 60.95)	25.90 (13.65, 44.45)	35.30 (15.18, 61.22)	33.50 (16.80, 56.10)	< 0.001
AI_NREM_ (events/h)	3.36 (0.43, 17.20)	1.24 (0.18, 5.40)	2.2 (0.17, 26.48)	2.41 (0.38, 14.95)	< 0.001
AI_REM_ (events/h)	5.56 (0.00, 27.33)	4.57 (0.00, 22.00)	11.69 (0.77, 36.00)	6.79 (0.00, 27.07)	0.113
HI_NREM_ (events/h)	16.29 (9.39, 25.32)	13.33 (7.67, 22.06)	10.73 (7.03, 21.62)	14.02 (8.37, 20.15)	0.019
HI_REM_ (events/h)	15.27 (6.63, 24.00)	15.00 (6.83, 24.00)	13.82 (8.00, 24.00)	14.88 (6.13, 25.58)	0.979
TST90 (%)	0.63 (0.03, 4.87)	0.18 (0.00, 1.36)	0.42 (0.00, 5.41)	0.40 (0.01, 3.95)	0.002
Mean oxygen desaturation (%)	4.00 (4.00, 5.00)	4.00 (4.00, 5.00)	4.00 (3.00, 5.50)	4.00 (4.00, 6.00)	0.043
Lowest oxygen saturation during sleep (%)	85.00 (79.00, 89.00)	87.00 (83.00, 90.00)	86.00 (77.25, 90.00)	85.00 (74.00, 90.00)	0.001
Mean oxygen saturation during sleep (%)	94.00 (93.00, 96.00)	95.00 (94.00, 96.00)	94.00 (93.00, 96.00)	95.00 (93.00, 96.00)	0.016
Longest duration of apnea (s)	52.00 (37.00, 66.00)	46.00 (30.25, 61.75)	52.00 (29.50, 60.50)	51.00 (34.00, 66.00)	0.021
Mean duration of apnea (s)	27.00 (21.00, 32.00)	25.00 (19.00, 31.00)	26.00 (19.00, 30.00)	25.00 (19.00, 31.00)	0.279
Longest duration of hypopnea (s)	77.00 (65.00, 89.00)	77.00 (64.00, 88.00)	66.50 (57.25, 78.75)	71.00 (62.00, 84.00)	0.004
Mean duration of hypopnea (s)	31.00 (27.00, 36.00)	32.00 (28.00, 35.50)	29.00 (26.00, 31.00)	30.00 (27.00, 34.00)	0.003
3. Limb movements and heart rate					
PLM index of sleep (events/h)	4.05 (0.80, 12.38)	2.00 (0.00, 10.12)	3.20 (0.30, 8.35)	5.40 (2.20, 15.25)	0.010
PLM index: NREM (events/h)	2.90 (0.45, 12.50)	2.20 (0.00, 11.95)	3.20 (0.00, 10.45)	5.50 (1.10, 16.90)	0.019
PLM index: REM (events/h)	0.00 (0.00, 2.55)	0.00 (0.00, 0.90)	0.00 (0.00, 3.38)	0.00 (0.00, 5.00)	0.015
Mean HR during sleep (bpm)	63.00 (59.00, 69.50)	62.00 (58.00, 67.00)	62.00 (58.00, 69.75)	63.00 (58.00, 70.00)	0.256
Highest HR during sleep (bpm)	94.37 ± 12.11	91.12 ± 11.44	97.28 ± 13.76	95.42 ± 10.68	< 0.001
Lowest HR during sleep (bpm)	49.00 (44.00, 53.00)	49.00 (45.00, 53.00)	46.50 (42.00, 52.75)	47.00 (44.00, 52.00)	0.089
Arrhythmia (%)	20 (8.5%)	20 (5.2%)	8 (14.8%)	8 (7.1%)	0.051

Continuous variables are presented as mea*n* ± standard deviation (SD) or median (25th, 75th percentiles), as appropriate. Categorical variables without missing observations are presented as *n* (%).

EDS, excessive daytime sleepiness; REM, rapid eye movement; NREM, non-rapid eye movement; N1%, percentage of N1 relative to total sleep time; N2%, percentage of N2 relative to total sleep time; N3%, percentage of N3 relative to total sleep time; REM%, percentage of REM relative to total sleep time; WASO, wake after sleep onset (min); LM, limb movement; PLM, periodic limb movement; AHI, apnea-hypopnea index; ODI, oxygen desaturation index; AI, apnea index; HI, hypopnea index; AHI_NREM_, AHI during NREM sleep; AHI_REM_, AHI during REM sleep; AHI_nonsupine_, AHI in non-supine position; AHI_supine_, AHI in supine position; AI_NREM_, apnea index during NREM sleep; AI_REM_, apnea index during REM sleep; HI_NREM_, hypopnea index during NREM sleep; HI_REM_, hypopnea index during REM sleep; TST90, percentage of total sleep time with SpO_2_ < 90%; HR, heart rate.

Respiratory-event burden also varied across symptom-groups. The AHI was lowest in poor sleep quality only and highest in EDS only, with median values of 18.50 and 28.43 events/h, respectively (*P* < 0.001). The ODI showed a similar pattern, with the lower values in poor sleep quality only and higher values in the EDS-related symptom-groups (*P* < 0.001). Several other hypoxemia-related indices, including TST90 and oxygen saturation parameters, also differed in unadjusted comparisons.

The composition of respiratory events also differed across symptom-groups. The apnea index (AI) varied significantly among the four symptom-groups, with a lower median value in poor sleep quality only than in EDS only (2.02 vs. 3.33 events/h; *P* = 0.001). In contrast, hypopnea events tended to be longer in poor sleep quality only, as the median longest hypopnea duration was 77.00 s in poor sleep quality only and 66.50 s in EDS only (*P* = 0.004), while mean hypopnea duration was also higher in poor sleep quality only (*P* = 0.003). Additional differences in sleep-stage variables, stage- or position-specific respiratory indices, limb movement parameters, and heart rate measures are presented in Table 2.

### 3.4 Factors associated with OSA symptom-groups

Given the number of candidate variables examined, the results are presented according to the strength and consistency of statistical support. All fitted models converged without evidence of complete or quasi-complete separation. Nevertheless, because the EDS-only group included only 54 participants and the model-specific complete-case sample was smaller for some predictors, estimates for this group were interpreted with particular attention to the width of the 95% CIs. Associations with nominal *P* values close to 0.05, wide CIs, or inconsistent sensitivity-analysis results were considered hypothesis-generating. Table 3 shows the results of the mutually adjusted demographic model and the separate multinomial logistic regression models for psychological and polysomnographic parameters. In the latter models, each parameter was entered individually, with adjustment for age, sex, and BMI. The low symptom burden group was used as the reference group in all analyses.

**Table 3:** Multinomial logistic regression analyses of factors associated with OSA symptom-groups.

Variables	Poor sleep quality only vs. Low symptom burden			EDS only vs. Low symptom burden			Combined vs. Low symptom burden		
	OR	95% CI	*P*	OR	95% CI	*P*	OR	95% CI	*P*
1. Demographic factors									
Age (per SD)	1.117	0.938–1.331	0.215	0.596	0.435–0.817	**0**.**001**	0.790	0.624–1.000	0.050
Sex, male	0.499	0.335–0.745	**<0**.**001**	0.613	0.290–1.295	0.200	0.664	0.380–1.161	0.151
BMI (per SD)	0.691	0.576–0.828	**<0**.**001**	1.296	0.985–1.703	0.064	1.139	0.912–1.422	0.252
2. Psychological factors									
GAD-7 (per SD)	2.192	1.719–2.795	**<0**.**001**	1.490	1.005–2.210	**0**.**047**	2.771	2.098–3.660	**<0**.**001**
PHQ-9 (per SD)	3.307	2.512–4.353	**<0**.**001**	3.081	2.099–4.521	**<0**.**001**	4.754	3.462–6.528	**<0**.**001**
CFS (per SD)	1.969	1.633–2.375	**<0**.**001**	2.243	1.601–3.141	**<0**.**001**	3.566	2.699–4.711	**<0**.**001**
3. Polysomnographic parameters									
3.1 Sleep stage and arousals									
Total sleep time (per SD)	0.829	0.694–0.990	**0**.**038**	1.281	0.918–1.789	0.146	1.028	0.808–1.309	0.822
Sleep efficiency (per SD)	0.840	0.697–1.012	0.066	1.362	0.916–2.024	0.127	0.985	0.761–1.274	0.907
Sleep onset latency (per SD)	1.420	1.127–1.790	**0**.**003**	0.124	0.036–0.429	**<0**.**001**	1.117	0.814–1.532	0.492
REM sleep latency (per SD)	1.041	0.879–1.232	0.645	0.690	0.481–0.990	**0**.**044**	0.871	0.684–1.108	0.262
N1% (per SD)	0.735	0.607–0.890	**0**.**002**	1.012	0.736–1.391	0.943	0.939	0.736–1.197	0.611
N2% (per SD)	1.328	1.115–1.582	**0**.**001**	0.990	0.727–1.349	0.950	0.992	0.788–1.250	0.948
N3% (per SD)	1.098	0.915–1.318	0.316	0.777	0.551–1.095	0.150	0.933	0.726–1.199	0.589
REM% (per SD)	1.018	0.852–1.216	0.843	1.422	1.042–1.942	**0**.**027**	1.314	1.034–1.670	**0**.**025**
Total arousal index (per SD)	0.858	0.714–1.032	0.103	1.005	0.753–1.341	0.974	0.924	0.732–1.166	0.505
Respiration-related arousal index (per SD)	0.791	0.653–0.959	**0**.**017**	1.043	0.793–1.373	0.762	0.907	0.721–1.143	0.409
Respiration-related arousal index: NREM (per SD)	0.780	0.643–0.946	**0**.**011**	1.041	0.793–1.367	0.770	0.907	0.721–1.140	0.402
Respiration-related arousal index: REM (per SD)	0.877	0.732–1.049	0.151	1.006	0.750–1.349	0.971	0.866	0.681–1.100	0.239
LM-related arousal index (per SD)	0.990	0.833–1.177	0.909	1.127	0.842–1.510	0.421	1.230	0.995–1.519	0.055
LM-related arousal index: REM (per SD)	1.249	1.012–1.542	**0**.**039**	1.302	0.973–1.741	0.076	1.264	0.985–1.620	0.065
PLM-related arousal index (per SD)	1.209	0.964–1.516	0.101	1.043	0.640–1.698	0.867	1.349	1.048–1.735	**0**.**020**
Spontaneous arousal index (per SD)	1.096	0.926–1.296	0.285	0.797	0.546–1.165	0.242	0.925	0.718–1.190	0.543
Spontaneous arousal index: NREM (per SD)	1.091	0.922–1.290	0.310	0.832	0.576–1.201	0.326	0.921	0.716–1.185	0.522
3.2 Parameters of OSA severity and hypoxemia									
AHI (per SD)	0.752	0.618–0.915	**0**.**004**	1.110	0.816–1.510	0.505	0.890	0.695–1.139	0.355
ODI (per SD)	0.815	0.669–0.992	**0**.**042**	1.162	0.855–1.578	0.338	0.974	0.763–1.245	0.835
AI (per SD)	0.824	0.680–0.999	**0**.**048**	1.305	0.998–1.708	0.052	1.060	0.848–1.325	0.610
HI (per SD)	0.830	0.697–0.988	**0**.**036**	0.723	0.524–0.998	**0**.**048**	0.741	0.583–0.943	**0**.**015**
AHI_NREM_ (per SD)	0.780	0.647–0.941	**0**.**009**	1.092	0.818–1.459	0.550	0.940	0.747–1.184	0.602
AHI_REM_ (per SD)	1.028	0.860–1.229	0.762	1.381	1.023–1.864	**0**.**035**	1.155	0.915–1.458	0.225
AHI_nonsupine_ (per SD)	0.861	0.710–1.043	0.126	1.353	1.024–1.789	**0**.**034**	1.066	0.846–1.342	0.590
AHI_supine_ (per SD)	0.815	0.670–0.991	**0**.**041**	1.012	0.734–1.396	0.940	0.927	0.720–1.193	0.555
AI_NREM_ (per SD)	0.776	0.628–0.958	**0**.**018**	1.224	0.933–1.606	0.144	0.995	0.790–1.253	0.965
HI_NREM_ (per SD)	0.829	0.690–0.997	**0**.**046**	0.719	0.514–1.006	0.054	0.741	0.578–0.950	**0**.**018**
TST90 (per SD)	0.896	0.741–1.082	0.254	1.076	0.829–1.397	0.580	1.067	0.869–1.309	0.535
Mean oxygen desaturation (per SD)	0.902	0.702–1.160	0.421	0.747	0.501–1.113	0.152	1.135	0.864–1.490	0.364
Lowest oxygen saturation during sleep (per SD)	1.261	1.042–1.525	**0**.**017**	1.001	0.743–1.349	0.993	0.937	0.748–1.174	0.570
Mean oxygen saturation during sleep (per SD)	1.124	0.925–1.365	0.239	0.924	0.694–1.230	0.586	0.967	0.767–1.219	0.776
Longest duration of apnea (per SD)	0.838	0.698–1.006	0.058	0.887	0.631–1.246	0.488	1.007	0.789–1.285	0.954
Mean duration of apnea (per SD)	0.901	0.753–1.078	0.256	0.908	0.649–1.269	0.570	0.957	0.744–1.230	0.731
Longest duration of hypopnea (per SD)	0.925	0.783–1.092	0.358	0.591	0.411–0.850	**0**.**005**	0.863	0.679–1.095	0.226
Mean duration of hypopnea (per SD)	0.978	0.817–1.170	0.806	0.700	0.485–1.011	0.057	0.929	0.720–1.197	0.567
3.3 Limb movements and heart rate									
PLM index of sleep (per SD)	1.071	0.897–1.280	0.448	0.947	0.638–1.407	0.789	1.270	1.020–1.581	**0**.**033**
PLM index: NREM (per SD)	1.101	0.919–1.320	0.298	0.929	0.616–1.401	0.726	1.302	1.043–1.626	**0**.**020**
PLM index: REM (per SD)	0.934	0.793–1.099	0.408	1.041	0.793–1.366	0.773	0.983	0.788–1.226	0.878
Highest HR during sleep (per SD)	0.810	0.674–0.974	**0**.**025**	1.004	0.721–1.397	0.981	0.986	0.771–1.261	0.911
Arrhythmia	0.611	0.316–1.183	0.144	1.953	0.788–4.841	0.148	0.850	0.360–2.011	0.712

The demographic model included age, sex, and BMI simultaneously. Each psychological or polysomnographic variable was entered separately into a multinomial logistic regression model adjusted for age, sex, and BMI. The low symptom burden group was defined as the reference group.

All continuous variables were Z-score standardized, and ORs for continuous variables are expressed per 1-SD increase.

*P* values were not adjusted for multiple comparisons. Nominally statistically significant results (two-sided *P* < 0.05) are shown in bold and should be interpreted together with the effect estimates, 95% CIs, and sensitivity analyses.

OSA, obstructive sleep apnea; EDS, excessive daytime sleepiness; OR, odds ratio; CI, confidence interval; SD, standard deviation; BMI, body mass index; GAD-7, Generalized Anxiety Disorder-7; PHQ-9, Patient Health Questionnaire-9; CFS, Chalder Fatigue Scale; REM, rapid eye movement; N1%, percentage of N1 relative to total sleep time; N2%, percentage of N2 relative to total sleep time; N3%, percentage of N3 relative to total sleep time; REM%, percentage of REM relative to total sleep time; NREM, non-rapid eye movement; LM, limb movement; PLM, periodic limb movement; AHI, apnea-hypopnea index; ODI, oxygen desaturation index; AI, apnea index; HI, hypopnea index; AHI_NREM_, AHI during NREM sleep; AHI_REM_, AHI during REM sleep; AHI_nonsupine_, AHI in non-supine position; AHI_supine_, AHI in supine position; AI_NREM_, apnea index during NREM sleep; HI_NREM_, hypopnea index during NREM sleep; TST90, percentage of total sleep time with SpO2 < 90%; HR, heart rate.

#### 3.4.1 Demographic correlates

In the demographic model, compared with the low symptom burden group, male sex and higher BMI were associated with lower odds of membership in the poor sleep quality only group (all *P* < 0.01). The OR for male sex was 0.499 (95% CI: 0.335-0.745), and the OR per SD increase in BMI was 0.691 (95% CI: 0.576-0.828). Conversely, younger age was the only demographic factor significantly associated with EDS only (OR per SD increase: 0.596, 95% CI: 0.435-0.817, *P* = 0.001). A similar inverse association was observed for the combined group, although the statistical significance was borderline (OR per SD increase: 0.790, 95% CI: 0.624-1.000, *P* = 0.050) and should be interpreted cautiously. No significant association of other demographic variables with any symptom-group was found (all *P* > 0.05).

#### 3.4.2 Psychological and fatigue-related correlates

For the psychological and fatigue-related measures, compared to those with low symptom burden, higher PHQ-9 and CFS scores were consistently associated with higher odds of membership in each of the three symptom groups (all *P* values <0.001). Higher GAD-7 scores were strongly associated with the poor sleep quality only and combined groups, with ORs per SD increase (95% CIs) being 2.192 (95% CI: 1.719–2.795) for the poor sleep quality only group and 2.771 (95% CI: 2.098–3.660) for the combined group, respectively (all *P* values <0.001), whereas the association with the EDS-only group was less precise and only nominally significant (OR per SD increase: 1.490, 95% CI: 1.005–2.210; *P* = 0.047).

#### 3.4.3 Polysomnographic correlates

Full results of polysomnographic parameters are shown in Table 3. In separate models adjusted for age, sex, and BMI, compared with the low symptom burden group, several associations were observed across measures of sleep stages and arousals, OSA severity and hypoxemia, and limb movements and heart rate.

Regarding sleep stages and arousals, poor sleep quality only was significantly associated with shorter total sleep time, longer sleep onset latency, a lower N1 sleep percentage, a higher N2 sleep percentage, and lower respiration-related arousal indices (all *P* < 0.05). In contrast, EDS only was characterized by markedly shorter sleep onset latency (OR per SD increase: 0.124, 95% CI: 0.036-0.429), shorter REM sleep latency (OR per SD increase: 0.690, 95% CI: 0.481-0.990), and a higher REM sleep percentage (OR per SD increase: 1.422, 95% CI: 1.042-1.942) (all *P* < 0.05). Also, a higher REM sleep percentage was associated with the combined symptom-group (OR per SD increase: 1.314, 95% CI: 1.034-1.670, *P* = 0.025). Several additional nominal associations involving arousal, limb-movement, and heart-rate measures are reported in Table 3 and should be considered hypothesis-generating.

For parameters of OSA severity and hypoxemia, higher overall and stage- or position-specific respiratory-event indices were generally associated with lower odds of poor sleep quality only, including AHI, ODI, apnea index, hypopnea index, NREM AHI, and supine AHI, with ORs per SD increase (95% CIs) being 0.752 (0.618–0.915), 0.815 (0.669-0.992), 0.824 (0.680–0.999), 0.830 (0.697–0.988), 0.780 (0.647–0.941), and 0.815 (0.670–0.991), respectively. A higher lowest oxygen saturation during sleep was also associated with poor sleep quality only (*P* = 0.017). The EDS-only group showed a different respiratory-event pattern, characterized by higher REM AHI (OR per SD increase: 1.381, 95% CI: 1.023-1.864) and non-supine AHI (OR per SD increase: 1.353, 95% CI: 1.024–1.789), a lower hypopnea index (OR per SD increase: 0.723, 95% CI: 0.524–0.998), and a shorter longest hypopnea duration (OR per SD increase: 0.591, 95% CI: 0.411–0.850) (all *P* < 0.05). The combined group was primarily associated with lower overall and NREM hypopnea indices. TST90, mean oxygen desaturation, and mean nocturnal oxygen saturation were not significantly associated with any symptom-group.

Regarding limb movements and heart rate, higher periodic limb movement indices during total sleep and NREM sleep were associated with the combined group, with ORs per SD increase (95% CIs) being 1.270 (1.020–1.581) and 1.302 (1.043–1.626), respectively (all *P* < 0.05), whereas a lower highest heart rate during sleep was associated with poor sleep quality only (OR per SD increase: 0.810, 95% CI: 0.674-0.974, *P* = 0.025). No significant associations were observed for arrhythmia or the remaining limb-movement and heart-rate parameters (all *P* > 0.05). Estimates for the EDS-only group should be interpreted with additional caution because of the relatively small number of participants in this group. Although several associations showed similar directions in the sensitivity analyses, the corresponding 95% CIs indicated limited precision for some estimates.

### 3.5 Sensitivity analysis restricted to moderate-to-severe OSA

Sensitivity analysis was performed after restricting the sample to patients with moderate-to-severe OSA. A total of 535 participants were included: 240 (44.9%) in the poor sleep quality only group, 176 (32.9%) in the low symptom burden group, 80 (15.0%) in the combined group, and 39 (7.3%) in the EDS-only group (Supplementary Table S1).

Descriptive comparisons showed similar differences in demographic characteristics, psychological symptom burden, sleep architecture, and respiratory-event profiles across the four symptom-groups (Supplementary Tables S1 and S2). The main directions of the adjusted associations were also largely preserved (Supplementary Table S3). The poor sleep quality only group remained associated with female sex, lower BMI, longer sleep latency, and higher PHQ-9 and GAD-7 scores. Associations between higher CFS scores and all three symptom-groups were also preserved in the moderate-to-severe OSA subgroup. The EDS-only group remained associated with an apnea-dominant respiratory profile, including higher apnea index and higher non-supine AHI, shorter longest hypopnea duration, and higher REM sleep percentage. The combined symptom-group retained strong associations with psychological symptom burden and higher REM percentage. Consistent with the absence of a uniform pattern in the primary analysis, none of the evaluated oxygen desaturation measures reached statistical significance in the moderate-to-severe OSA subgroup after adjustment for age, sex, and BMI. Estimates for the EDS-only group should be interpreted with additional caution because of the relatively small number of participants in this group. Although several associations showed similar directions in the sensitivity analyses, the corresponding 95% CIs indicated limited precision for some estimates.

### 3.6 Sensitivity analysis after excluding patients with any coexisting sleep disorder

After restricting the analysis to participants with available coexisting-sleep-disorder status and no documented coexisting sleep disorder, 538 participants were eligible for the sensitivity analysis, including 34 participants in the EDS-only group. Model-specific complete-case sample sizes were smaller for some predictors. The overall direction of the principal associations was generally consistent with that observed in the primary cohort, although several nominal associations were attenuated. In the combined group, the associations with PLM indices during total sleep and NREM sleep were attenuated, whereas the PLM-related arousal index remained nominally associated with group membership (OR per SD increase: 1.640, 95% CI: 1.064–2.528; *P* = 0.025) (Supplementary Table S4).

### 3.7 Sensitivity analysis of polysomnographic correlates with additional adjustment for AHI

After additional adjustment for AHI, the principal associations of the poor sleep quality only group with shorter total sleep time, longer sleep onset latency, and higher N2% persisted. The EDS-only group retained associations with shorter sleep and REM latencies, higher REM%, higher non-supine AHI, lower HI, and shorter maximum hypopnea duration. The combined group retained associations with higher REM%, lower HI and NREM HI, and higher PLM indices during total and NREM sleep. Several respiratory severity, arousal, and oxygenation associations in the poor sleep quality only group, including the associations of lower ODI and higher lowest SpO₂, were attenuated after additional adjustment for AHI. A nominal association between lower mean oxygen desaturation and the EDS-only group emerged only in this AHI-adjusted analysis (OR per SD increase: 0.626, 95% CI: 0.414–0.946; *P* = 0.026), whereas the association between REM-AHI and the EDS-only group was reduced to borderline significance. Stage- and position-specific respiratory indices in these models represent conditional differences at a comparable overall AHI (Supplementary Table S5).

## 4 Discussion

In this retrospective cross-sectional study of treatment-naïve patients with OSA, we observed substantial heterogeneity across four symptom-groups. The poor sleep quality only group was the most prevalent symptom-group, accounting for 49.0% of the overall population and remaining the dominant symptom-group even among patients with moderate-to-severe OSA. The four questionnaire-defined symptom-groups showed multidimensional profiles that were not captured by AHI-defined severity alone. Female sex, lower BMI, altered sleep continuity and sleep-stage distribution, and generally lower respiratory-event burden were associated with the poor sleep quality only group. In contrast, younger age, shorter sleep onset and REM sleep latencies, a higher REM sleep percentage, and higher REM and non-supine AHI were associated with the EDS only group. Higher anxiety and depressive symptom scores were associated with all three higher symptom-burden groups, with the strongest associations observed for the combined symptom-group, which was also associated with a higher REM sleep percentage and higher periodic limb movement indices. Several conventional hypoxemia measures, including TST90, mean oxygen desaturation, and mean nocturnal oxygen saturation, were not significantly associated with any symptom-group. Findings were generally consistent with moderate-to-severe OSA, while additional adjustment for AHI preserved several sleep continuity, sleep-stage, and limb-movement associations but attenuated some respiratory-event, arousal, and oxygenation associations.

### 4.1 Demographic factors associated with OSA symptom-groups: age, sex, and BMI

Our study found that age, sex, and BMI showed symptom-group-specific associations with OSA symptom expression in the mutually adjusted demographic model. Relative to the low symptom burden group, the poor sleep quality only symptom-group was associated with female sex and lower BMI. The EDS only symptom-group was associated with younger age and tended to be associated with higher BMI. The combined symptom-group was also marginally associated with younger age. These findings are broadly consistent with previous studies showing demographic heterogeneity across OSA symptom-groups, although the direction and magnitude of these associations varied across studies (16, 19, 23).

The higher proportion of women in the poor sleep quality only symptom-group aligns with existing evidence that insomnia is more common in women than in men (29). This pattern may be related to sex differences in sleep structure, vulnerability to hyperarousal, and symptom perception and reporting. The association between younger age and EDS is also consistent with previous epidemiological observations (30, 31). Several mechanisms may explain why older individuals report less subjective sleepiness despite objectively measured OSA. These include reduced sensitivity to sleep fragmentation or intermittent hypoxemia, greater opportunities for daytime napping after retirement, and age-related changes in sleep need and homeostatic sleep regulation (31, 32)^.^

Although the adjusted association between higher BMI and EDS-only did not reach nominal statistical significance (*P* = 0.064), patients with EDS-only had the highest median BMI in the descriptive comparisons. This finding is concordant with prior evidence that obesity may contribute to subjective daytime sleepiness (33–35). Potential mechanisms include systemic inflammation, involving cytokines such as interleukin-6 and tumor necrosis factor-α, and metabolic dysregulation that may occur independently of sleep-disordered breathing severity (36). Longitudinal studies further suggest that obesity and weight gain are associated with the development and persistence of EDS, whereas weight loss is associated with its remission (37, 38). These findings suggest that weight status may remain relevant when evaluating sleepiness-predominant OSA.

### 4.2 Polysomnographic characteristics across OSA symptom-groups: OSA severity, respiratory event characteristics, and sleep architecture

We observed associations between several polysomnographic characteristics and OSA symptom-groups after adjustment for age, sex, and BMI. However, the strength of statistical support varied across measures, and these findings were interpreted jointly with the sensitivity analysis. Regarding OSA severity, higher AHI was associated with lower odds of the poor sleep quality only symptom-group relative to the low symptom burden group, but was not associated with the EDS only or combined groups. This finding is consistent with previous studies suggesting that AHI alone does not fully capture the clinical and symptomatic heterogeneity of OSA (12, 13, 39). Notably, TST90, mean oxygen desaturation, and mean nocturnal SpO₂ were not significantly associated with any questionnaire-defined symptom-group in the primary analysis. Although lower ODI and higher lowest SpO₂ were nominally associated with the poor sleep quality only group, these associations were attenuated after additional adjustment for overall AHI and did not reach conventional statistical significance in the moderate-to-severe OSA subgroup. Conversely, a nominal association between lower mean oxygen desaturation and the EDS-only group emerged only after additional adjustment for AHI. Taken together, conventional summary measures of nocturnal oxygenation did not show a consistent pattern across the questionnaire-defined symptom groups.

This lack of a consistent pattern does not establish that nocturnal hypoxemia is unrelated to symptom expression. Rather, it suggests that symptom heterogeneity in this cohort was not explained simply by the severity of hypoxemia captured by these conventional indices. More comprehensive measures incorporating the frequency, depth, and duration of oxygen desaturation, such as hypoxic burden, may provide different physiological information and warrant evaluation in future prospective studies.

With regard to respiratory event characteristics, higher REM AHI and non-supine AHI, a lower hypopnea index, and a shorter longest hypopnea duration were associated with higher odds of the EDS only symptom-group relative to the low symptom burden group. This stage- and position-specific pattern suggests that the distribution and characteristics of respiratory events may provide information about subjective sleepiness beyond overall event frequency alone. Differences in upper-airway vulnerability across sleep stages and body positions may partly contribute to this pattern (40, 41). However, physiological traits such as critical closing pressure, arousal threshold, loop gain, and upper-airway muscle responsiveness were not directly measured in this study. This mechanistic interpretation should therefore be regarded as hypothesis-generating rather than definitive.

Sleep architecture also showed symptom-specific associations. A higher REM sleep percentage was associated with higher odds of the EDS only and combined symptom-groups. Although a higher proportion of REM sleep is not equivalent to REM-predominant OSA, this association is biologically plausible because the upper airway is particularly vulnerable to collapse during REM sleep. REM-related OSA may also be accompanied by substantial daytime sleepiness despite a relatively low overall AHI (42, 43). In contrast, the poor sleep quality only symptom-group showed altered sleep continuity and sleep-stage distribution, characterized by shorter total sleep time, prolonged sleep onset latency, a lower proportion of N1 sleep, a higher proportion of N2 sleep, and lower respiration-related arousal indices. This pattern is broadly consistent with previous research showing disrupted sleep continuity and altered sleep architecture in patients with coexisting insomnia symptoms and OSA (11, 44). However, stage N3 percentage was not associated with any symptom-group after adjustment, suggesting that slow-wave sleep was not a major feature distinguishing these symptom-groups. The combined symptom-group was also associated with higher periodic limb movement indices during total and NREM sleep, although these exploratory findings should be interpreted cautiously.

Taken together, the primary and AHI-adjusted analyses suggest that symptom-group differences were not fully captured by overall respiratory-event frequency. Associations with sleep continuity, REM sleep percentage, and PLM indices generally persisted, whereas several respiratory-event, arousal, and oxygenation associations, particularly in the poor sleep quality only group, were attenuated, indicating that some primary findings reflected overall OSA severity. In the EDS-only group, associations with non-supine AHI, lower HI, and shorter maximum hypopnea duration persisted, whereas the association with REM-AHI became borderline. Because stage- and position-specific AHI and apnea or hypopnea indices were evaluated conditional on overall AHI, these estimates reflect respiratory-event distribution or composition at a comparable total event burden. Thus, detailed polysomnographic measures may complement overall AHI in characterizing OSA symptom heterogeneity. Accordingly, greater interpretive weight should be placed on associations supported by smaller *P* values and consistent results across the primary and sensitivity analyses. Several sleep-stage, stage- or position-specific respiratory, limb-movement, and heart-rate associations were supported only by nominal *P* values or were attenuated in sensitivity analyses and should therefore be considered hypothesis-generating.

### 4.3 Psychological symptom burden across OSA symptom-groups

Our study found that higher PHQ-9 and CFS scores were consistently associated with all three symptom-groups relative to the low symptom burden group after adjusting for age, sex, and BMI. Higher GAD-7 scores were also strongly associated with the poor sleep quality only and combined groups, whereas the association with the EDS-only group was less precise and only nominally significant. The combined group showed the greatest overall psychological and fatigue-related symptom burden. These findings are broadly consistent with a previous study reporting greater depressive symptom burden in the insomnia-related and combined insomnia-sleepiness OSA phenotypes, although no significant association was observed for the sleepiness-only phenotype (45).

These associations should be interpreted in light of the conceptual overlap among poor sleep quality, daytime sleepiness, fatigue, and psychological symptoms. These constructs are clinically distinguishable but are not entirely independent. The PSQI includes a daytime dysfunction component, the PHQ-9 includes sleep- and energy-related items, and the fatigue measured by the CFS may partially overlap with, although it is not equivalent to, the propensity to fall asleep assessed by the ESS. Because these measures were collected concurrently using self-report questionnaires, shared symptom content and common-method reporting may have contributed to some of the observed associations.

Previous studies of comorbid insomnia and OSA have reported greater dysfunctional sleep-related beliefs, presleep arousal, and anxiety and depressive symptoms (11, 46), and the hyperarousal model has been proposed as a framework for understanding insomnia symptoms (47). Associations between depressive symptoms and EDS in patients with OSA have also been reported (48, 49). However, the present questionnaire-defined grouping did not establish a diagnosis of insomnia, and the cross-sectional design precludes determination of the temporal or causal direction of the observed associations. The data cannot distinguish whether psychological symptoms preceded sleep-related symptom reporting, followed persistent sleep-related symptoms, or reflected shared underlying factors or overlapping symptom measurement. The findings should therefore be interpreted as evidence of co-occurring psychological, fatigue-related, and sleep-related symptom burden rather than evidence that psychological symptoms caused membership in a particular OSA symptom-group.

### 4.4 Cardiometabolic characteristics across OSA symptom-groups

In the descriptive analysis, the prevalence of cardiovascular disease showed a nominal difference across the four symptom groups, whereas the prevalence of hypertension and diabetes did not differ significantly. This finding was based on an unadjusted comparison of prevalent comorbidities and should be interpreted cautiously, particularly given the relatively small number of cardiovascular disease events. It does not establish that the symptom groups differ in their subsequent cardiovascular risk. Prospective studies are needed to determine whether questionnaire-defined symptom groups provide prognostic information beyond established cardiovascular risk factors and conventional measures of OSA severity. Previous studies have reported inconsistent findings regarding phenotype-specific cardiometabolic risk. Some observed a greater burden of cardiometabolic comorbidities in insomnia-related phenotypes, whereas others linked excessively sleepy phenotypes to increased cardiovascular events or mortality (16–21). These discrepancies may reflect differences in study populations, symptom definitions, outcome measures, and adjustment strategies. However, because the present study was cross-sectional, the absence of differences in baseline cardiometabolic characteristics should not be interpreted as evidence of equivalent long-term cardiovascular risk across symptom-groups.

### 4.5 Clinical implications

Our findings highlight the importance of combining symptom profiles with a broader polysomnographic profile, rather than relying solely on AHI or conventional oxygenation measures in clinical evaluation of OSA, because patients with similar physiological severity may experience markedly different symptom burdens. In addition, patients reporting prominent sleep-quality complaints or symptoms suggestive of insomnia should undergo further clinical assessment to determine whether an insomnia disorder is present. If insomnia disorder is confirmed, CBT-I may be considered alongside OSA-directed treatment (50, 51). Moreover, psychological symptom burden was higher across the other three symptom groups relative to the low symptom burden group, particularly in the combined group. Routine assessment of psychological distress may therefore help characterize co-occurring psychological symptom burden and inform appropriate referral or multidisciplinary care. Nevertheless, the symptom-specific polysomnographic patterns identified in our study are not yet sufficient to guide treatment selection. Prospective studies are needed to determine whether these questionnaire-defined symptom groups are associated with differences in treatment adherence or response.

### 4.6 Strengths and limitations

This study has several strengths. First, we included a relatively large cohort of consecutive, treatment-naïve patients with OSA who underwent standardized overnight polysomnography. Second, the PSQI- and ESS-based symptom classification provided a simple and transparent rule-based grouping strategy. Third, the integration of detailed polysomnographic parameters and psychological assessments, together with covariate-adjusted models and sensitivity analyses, allowed a multidimensional and more comprehensive characterization of OSA symptom heterogeneity. However, several limitations should also be acknowledged. First, participants were recruited from a single tertiary sleep center, which may have introduced referral or selection bias and limit the generalizability of the findings to broader community-based OSA populations. Second, the retrospective cross-sectional design precluded assessment of temporal relationships, longitudinal outcomes, or treatment responses; therefore, causality between the identified factors and specific symptom-groups cannot be inferred. Third, symptom-group classification relied on self-reported PSQI and ESS scores and was therefore subject to recall and reporting bias. The PSQI does not establish a diagnosis of insomnia, and the >7 cutoff, although supported by validation in a Chinese population, may limit comparisons with studies using >5. In addition, reducing symptom information to PSQI and ESS thresholds may not capture broader latent symptom structures. The classification should therefore be interpreted as an operational grouping of two questionnaire dimensions rather than evidence of biologically distinct OSA subtypes. Furthermore, the PSQI, ESS, PHQ-9, GAD-7, and CFS are self-reported measures of related symptom domains that are not entirely independent. Conceptual overlap—particularly among poor sleep quality, daytime sleepiness, fatigue, and depressive symptoms—as well as common-method reporting may therefore have contributed to some of the observed associations. Fourth, as the present study was observational, residual confounding could not be completely ruled out; moreover, intrinsic pathophysiological traits such as critical closing pressure, loop gain, and arousal threshold were not directly measured. Fifth, the large number of candidate polysomnographic and questionnaire variables examined without formal adjustment for multiple comparisons increased the possibility of chance findings. Nominal associations, particularly those with *P* values close to 0.05, CIs close to the null, or inconsistent sensitivity-analysis results, should therefore be regarded as exploratory and require independent replication. Sixth, the unequal sample sizes across symptom-groups, particularly the relatively small EDS-only group, may limit the precision of some estimates. The number of EDS-only participants was further reduced in models with predictor-specific missing data and in the sensitivity-analysis subsets. Although the fitted models converged and several associations showed similar directions across sensitivity analyses, these findings do not fully overcome the uncertainty associated with the small EDS-only group. Estimates with wide 95% CIs or nominal *P* values close to 0.05 should therefore be interpreted cautiously and require confirmation in larger cohorts. Future prospective, multicenter studies incorporating longitudinal follow-up and direct physiological measurements are warranted to validate these findings and clarify the underlying endotypic mechanisms.

## 5 Conclusion

Questionnaire-defined OSA symptom groups differed in demographic, polysomnographic, and psychological characteristics. Findings with stronger and more consistent statistical support included female sex and lower BMI in the poor sleep quality only group, younger age and shorter sleep onset latency in the EDS-only group, and higher PHQ-9 and CFS scores across all three symptom groups relative to the low symptom burden group. The largest effect estimates for psychological and fatigue-related measures were observed in the combined group. Several sleep-stage, stage- or position-specific respiratory, limb-movement, and heart-rate associations were nominal and should be regarded as hypothesis-generating. Overall, the findings support multidimensional clinical assessment beyond AHI alone, but prospective studies and independent replication are needed to determine the stability and clinical relevance of these associations.

## Data Availability

The raw data supporting the conclusions of this article will be made available by the authors, without undue reservation.
