# Supplementary material for: Clinical, polysomnographic, and psychological profiles across questionnaire-defined symptom groups in obstructive sleep apnea
Source: Front Med (Lausanne). 2026 Sep 30;13:1948049. doi: 10.3389/fmed.2026.1948049 (PMC13662046; doi:10.3389/fmed.2026.1948049)
Supplement: Supplementary file 1 [file Table1.docx]

***Supplementary Material***

**Supplementary Tables**

**Supplementary Table 1** General characteristics of the four symptom-groups in moderate-to-severe OSA.

| **Characteristics** | **Symptom-groups** | | | | ***P*** |
| --- | --- | --- | --- | --- | --- |
|  | **Low symptom burden** | **Poor sleep quality only** | **EDS only** | **Combined** |  |
| Number | 176 | 240 | 39 | 80 |  |
| Sex, male, n (%) | 145 (82.4%) | 167 (69.6%) | 33 (84.6%) | 65 (81.2%) | **0.007** |
| Age (years) | 51.00 (41.00, 62.00) | 55.00 (45.00, 64.00) | 49.00 (30.00, 63.00) | 49.50 (36.00, 58.00) | **0.001** |
| BMI (kg/m^2^) | 26.50 (23.87, 28.39) | 25.07 (22.85, 27.26) | 26.49 (24.70, 29.40) | 26.40 (24.46, 29.92) | **<0.001** |
| PSQI | 5.00 (3.00, 6.00) | 11.00 (9.00, 15.00) | 6.00 (4.50, 7.00) | 11.00 (9.75, 12.00) | - |
| ESS | 4.00 (3.00, 6.00) | 3.00 (1.75, 6.00) | 12.00 (11.00, 16.50) | 13.00 (11.00, 15.00) | - |
| PHQ-9 | 2.00 (0.00, 4.00) | 6.00 (3.00, 8.00) | 3.00 (1.50, 9.00) | 8.00 (4.00, 11.00) | **<0.001** |
| GAD-7 | 1.00 (0.00, 4.00) | 4.00 (2.00, 7.00) | 3.00 (0.00, 6.00) | 5.00 (3.00, 8.00) | **<0.001** |
| CFS | 5.00 (2.00, 7.00) | 7.00 (5.00, 9.00) | 8.00 (5.50, 9.00) | 9.00 (7.00, 10.50) | **<0.001** |
| Any coexisting sleep disorder, n/N (%)^a^ | 25/156 (16.0%) | 38/215 (17.7%) | 7/36 (19.4%) | 13/75 (17.3%) | 0.958 |
| Hypertension, n (%) | 78 (44.3%) | 105 (43.8%) | 16 (41.0%) | 27 (33.8%) | 0.404 |
| Diabetes, n (%) | 20 (11.4%) | 33 (13.8%) | 4 (10.3%) | 7 (8.8%) | 0.641 |
| Cardiovascular disease, n (%)^b^ | 31 (17.6%) | 25 (10.4%) | 5 (12.8%) | 9 (11.2%) | 0.178 |

Continuous variables are presented as median (25^th^, 75^th^ percentiles). Categorical variables with missing observations are presented as n/N (%), where N represents the number of participants with available data; those without missing observations are presented as n (%), and percentages were calculated using the total number of participants in each symptom-group.

OSA, obstructive sleep apnea; EDS, excessive daytime sleepiness; BMI, body mass index; PSQI, Pittsburgh Sleep Quality Index; ESS, Epworth Sleepiness Scale; PHQ-9, Patient Health Questionnaire-9; GAD-7, Generalized Anxiety Disorder-7; CFS, Chalder Fatigue Scale.

Statistically significant results (*P* < 0.05) are shown in **bold.**

^a^  Coexisting sleep disorders included restless legs syndrome, REM sleep behavior disorder, and narcolepsy.

^b^ Cardiovascular disease (including myocardial infarction, coronary artery disease, congestive heart failure, and stroke).

PSQI and ESS were used to define the four symptom-groups. Their distributions are therefore presented for descriptive purposes only, and the corresponding between-group P values are not reported.

**Supplementary Table 2** Polysomnographic parameters across the four symptom-groups in moderate-to-severe OSA.

| **Characteristics** | **Symptom-groups** | | | | ***P*** |
| --- | --- | --- | --- | --- | --- |
|  | **Low symptom burden** | **Poor sleep quality only** | **EDS only** | **Combined** |  |
| **Number** | 176 | 240 | 39 | 80 |  |
| **1. Sleep stages and arousals** | | | | | |
| Total sleep time (min) | 415.00  (351.25, 456.25) | 387.25  (326.50, 442.62) | 407.00  (379.75, 469.75) | 416.00  (347.75, 459.75) | **0.006** |
| Sleep efficiency (%) | 77.70  (66.97, 84.03) | 74.15  (62.27, 81.53) | 81.30  (69.30, 90.60) | 75.80  (64.78, 85.43) | **0.001** |
| Sleep latency (min) | 8.00 (5.50, 16.12) | 11.00 (5.88, 19.75) | 4.00 (1.75, 7.75) | 6.75 (3.38, 15.25) | **<0.001** |
| REM sleep latency (min) | 103.50  (75.50, 162.12) | 110.75  (79.38, 179.00) | 91.00  (56.75, 123.75) | 107.25  (63.12, 152.88) | **0.041** |
| N1% (%) | 36.15  (26.50, 50.47) | 30.35  (23.65, 40.90) | 29.30  (22.15, 49.65) | 34.15  (23.68, 45.85) | **0.012** |
| N2% (%) | 34.72 ± 10.53 | 38.17 ± 10.68 | 35.56 ± 12.40 | 34.88 ± 11.61 | **0.007** |
| N3% (%) | 11.60  (5.22, 17.60) | 13.20  (7.65, 20.62) | 11.00  (3.30, 17.70) | 10.50  (4.17, 18.40) | **0.037** |
| REM% (%) | 13.70  (9.80, 17.70) | 13.85  (10.47, 17.40) | 16.30  (13.20, 18.65) | 15.00  (11.60, 20.05) | **0.015** |
| WASO (min) | 107.00  (78.50, 159.50) | 118.75  (81.88, 181.75) | 86.50  (45.50, 149.00) | 121.00  (74.00, 175.62) | 0.057 |
| Index of sleep stage transformation (events/h) | 44.88  (36.93, 60.13) | 43.68  (38.12, 54.27) | 36.93  (31.01, 50.02) | 41.39  (33.20, 55.23) | 0.749 |
| Index of sleep to wake transitions (events/h) | 6.73 (4.77, 8.92) | 6.68 (4.97, 8.40) | 5.69 (3.56, 9.21) | 6.83 (4.46, 8.51) | 0.520 |
| Total arousal index (events/h) | 37.60  (26.77, 54.05) | 33.60  (25.38, 45.17) | 32.70  (26.30, 61.00) | 33.65  (26.05, 49.12) | 0.076 |
| Respiration-related arousal index (events/h) | 22.75  (14.65, 40.97) | 20.15  (13.78, 29.85) | 21.10  (15.20, 50.00) | 21.65  (13.15, 34.05) | **0.044** |
| Respiration-related arousal index: NREM (events/h) | 22.70  (14.80, 42.20) | 20.15  (13.47, 30.82) | 21.70  (15.65, 52.20) | 21.65  (12.65, 39.33) | **0.035** |
| Respiration-related arousal index: REM (events/h) | 21.95  (11.40, 35.68) | 19.70  (11.62, 30.90) | 21.20  (10.10, 44.00) | 20.05  (11.78, 32.85) | 0.308 |
| LM-related arousal index (events/h) | 0.30 (0.10, 0.60) | 0.30 (0.00, 0.50) | 0.30 (0.10, 0.55) | 0.30 (0.00, 0.72) | 0.476 |
| LM-related arousal index: NREM (events/h) | 0.30 (0.10, 0.70) | 0.30 (0.00, 0.60) | 0.40 (0.15, 0.60) | 0.30 (0.00, 0.70) | 0.275 |
| LM-related arousal index: REM (events/h) | 0.00 (0.00, 0.00) | 0.00 (0.00, 0.00) | 0.00 (0.00, 0.30) | 0.00 (0.00, 0.00) | 0.299 |
| PLM-related arousal index (events/h) | 0.10 (0.00, 0.60) | 0.00 (0.00, 0.60) | 0.00 (0.00, 1.00) | 0.20 (0.00, 1.05) | 0.231 |
| PLM-related arousal index: NREM (events/h) | 0.20 (0.00, 0.70) | 0.00 (0.00, 0.70) | 0.00 (0.00, 1.15) | 0.20 (0.00, 0.95) | 0.242 |
| PLM-related arousal index: REM (events/h) | 0.00 (0.00, 0.00) | 0.00 (0.00, 0.00) | 0.00 (0.00, 0.00) | 0.00 (0.00, 0.00) | 0.058 |
| Spontaneous arousal index (events/h) | 10.10 (7.57, 14.33) | 10.30 (7.65, 14.95) | 8.30 (6.65, 12.00) | 9.05 (6.52, 12.80) | **0.016** |
| Spontaneous arousal index: NREM (events/h) | 10.80 (7.70, 15.40) | 11.10 (7.88, 15.40) | 8.20 (6.60, 13.05) | 9.85 (6.30, 13.12) | **0.015** |
| Spontaneous arousal index: REM (events/h) | 6.65 (3.30, 10.28) | 6.85 (4.10, 10.93) | 7.30 (3.95, 9.35) | 6.95 (4.35, 9.77) | 0.691 |
| **2. Parameters of OSA severity and hypoxemia** | | | | | |
| AHI (events/h) | 33.58  (24.02, 57.50) | 29.44  (19.90, 43.53) | 39.50  (24.74, 65.29) | 35.72  (23.76, 53.64) | **<0.001** |
| ODI (events/h) | 25.45  (12.07, 46.12) | 20.10  (9.47, 33.77) | 29.60  (14.50, 63.10) | 24.00  (16.50, 44.08) | **0.002** |
| AI (events/h) | 7.97  (3.13, 24.54) | 5.97  (1.77, 16.92) | 18.54  (2.87, 43.77) | 11.89  (1.86, 26.85) | **0.005** |
| HI (events/h) | 20.60  (14.51, 28.03) | 18.75  (14.42, 26.29) | 16.61  (11.89, 23.80) | 17.72  (14.40, 23.57) | 0.119 |
| AHI_NREM_ (events/h) | 29.21  (18.58, 52.38) | 24.12  (16.60, 37.27) | 33.36  (20.88, 56.90) | 29.66  (18.40, 46.31) | **0.007** |
| AHI_REM_ (events/h) | 26.90  (16.62, 43.45) | 28.70  (16.00, 43.73) | 46.50  (15.20, 59.95) | 33.45  (18.48, 55.07) | **0.016** |
| AHI_nonsupine_ (events/h) | 40.10  (17.60, 76.65) | 32.05  (11.95, 59.98) | 67.80  (42.45, 105.05) | 44.30  (23.18, 81.28) | **<0.001** |
| AHI_supine_ (events/h) | 46.60  (29.80, 64.95) | 37.50  (26.08, 57.00) | 49.60  (33.10, 65.10) | 46.20  (31.27, 63.88) | **0.005** |
| AI_NREM_ (events/h) | 7.39  (2.10, 27.63) | 3.48  (0.90, 15.29) | 12.67  (1.67, 44.77) | 8.16  (1.25, 25.94) | **0.002** |
| AI_REM_ (events/h) | 14.56  (1.82, 33.66) | 14.44  (2.12, 29.15) | 26.35  (8.00, 43.96) | 19.02  (2.74, 31.90) | 0.189 |
| HI_NREM_ (events/h) | 20.14  (14.66, 28.57) | 19.25  (14.00, 26.63) | 16.98  (9.92, 26.06) | 17.61  (13.58, 22.97) | 0.162 |
| HI_REM_ (events/h) | 16.93  (7.08, 25.73) | 18.75  (10.02, 28.05) | 16.52  (6.62, 28.26) | 16.70  (9.01, 28.39) | 0.621 |
| TST90 (%) | 1.84 (0.18, 7.12) | 0.65 (0.09, 2.61) | 1.67 (0.24, 8.30) | 2.14 (0.29, 5.78) | **0.001** |
| Average oxygen desaturation (%) | 5.00 (4.00, 6.00) | 4.00 (4.00, 5.00) | 4.00 (3.50, 6.50) | 5.00 (4.00, 7.00) | **0.071** |
| Lowest oxygen saturation during sleep (%) | 83.00  (76.00, 88.00) | 85.00  (80.00, 88.00) | 83.00  (71.00, 88.00) | 84.00  (69.00, 86.00) | **0.001** |
| Average oxygen saturation during sleep (%) | 94.00  (92.00, 95.00) | 94.00  (93.00, 95.00) | 94.00  (91.50, 95.00) | 94.00  (92.00, 95.00) | **0.032** |
| Longest duration of apnea (s) | 56.00  (45.00, 68.00) | 52.00  (40.00, 67.75) | 57.00  (43.00, 62.00) | 59.00  (42.00, 72.00) | 0.346 |
| Mean duration of apnea (s) | 28.00  (23.75, 33.00) | 27.00  (23.00, 33.00) | 28.00  (22.50, 30.00) | 27.00  (23.75, 33.00) | 0.786 |
| Longest duration of hypopnea (s) | 76.00  (63.00, 89.00) | 77.00  (65.75, 88.25) | 66.00  (56.50, 82.00) | 71.50  (61.75, 85.25) | **0.030** |
| Mean duration of hypopnea (s) | 30.00  (27.00, 35.00) | 31.00  (27.75, 35.00) | 29.00  (26.50, 31.50) | 30.00  (27.00, 33.25) | **0.050** |
| **3. Limb movements and heart rate** | | | | | |
| PLM index of sleep (events/h) | 5.00 (1.00, 11.60) | 2.50 (0.18, 10.10) | 3.60 (0.45, 8.20) | 6.30 (1.77, 15.55) | 0.082 |
| PLM index: NREM (events/h) | 3.70 (0.70, 12.45) | 2.40 (0.28, 11.62) | 4.20 (0.00, 11.10) | 6.00 (1.00, 16.97) | 0.145 |
| PLM index: REM (events/h) | 0.00 (0.00, 2.52) | 0.00 (0.00, 0.90) | 0.00 (0.00, 3.45) | 0.00 (0.00, 5.20) | 0.067 |
| Average HR during sleep (bpm) | 64.00  (59.00, 70.00) | 63.00  (58.00, 67.00) | 63.00  (57.50, 71.50) | 63.00  (59.00, 70.25) | 0.248 |
| Highest HR during sleep (bpm) | 94.90 ± 11.83 | 91.94 ± 11.34 | 97.87 ± 13.37 | 95.29 ± 10.81 | **0.003** |
| Lowest HR during sleep (bpm) | 49.00  (44.00, 53.00) | 49.00  (45.00, 53.00) | 46.00  (42.00, 53.00) | 47.00  (43.00, 52.00) | 0.135 |
| Arrhythmia (%) | 17 (9.7%) | 15 (6.2%) | 6 (15.4%) | 7 (8.8%) | 0.232 |

Continuous variables are presented as mean ± standard deviation (SD) or median (25^th^, 75^th^ percentiles), as appropriate. Categorical variables without missing observations are presented as n (%).

EDS, excessive daytime sleepiness; REM, rapid eye movement; NREM, non-rapid eye movement; N1%, percentage of N1 relative to total sleep time; N2%, percentage of N2 relative to total sleep time; N3%, percentage of N3 relative to total sleep time; REM%, percentage of REM relative to total sleep time; WASO, wake after sleep onset (min); LM, limb movement; PLM, periodic limb movement; AHI, apnea-hypopnea index; ODI, oxygen desaturation index; AI, apnea index; HI, hypopnea index; AHI_NREM_, AHI during NREM sleep; AHI_REM_, AHI during REM sleep; AHI_nonsupine_, AHI in non-supine position; AHI_supine_, AHI in supine position; AI_NREM_, apnea index during NREM sleep; AI_REM_, apnea index during REM sleep; HI_NREM_, hypopnea index during NREM sleep; HI_REM_, hypopnea index during REM sleep; TST90, percentage of total sleep time with SpO_2_ < 90%; HR, heart rate.

Statistically significant results (*P* < 0.05) are shown in **bold.**

**Supplementary Table 3** Multinomial logistic regression analyses of factors associated with OSA symptom-groups among patients with moderate-to-severe OSA.

| **Variable** | **Poor sleep quality only vs Low symptom burden** | | | **EDS only vs Low symptom burden** | | | **Combined vs Low symptom burden** | | |
| --- | --- | --- | --- | --- | --- | --- | --- | --- | --- |
|  | **OR** | **95% CI** | ***P*** | **OR** | **95% CI** | ***P*** | **OR** | **95% CI** | ***P*** |
| **1. Demographic factors** | | | | | | | | | |
| Age (per SD) | 1.091 | 0.876-1.359 | 0.436 | 0.738 | 0.502-1.083 | 0.121 | 0.822 | 0.613-1.102 | 0.190 |
| Sex, male | 0.569 | 0.339-0.955 | **0.033** | 0.843 | 0.305-2.330 | 0.742 | 0.724 | 0.347-1.510 | 0.389 |
| BMI (per SD) | 0.681 | 0.547-0.848 | **<0.001** | 1.159 | 0.818-1.641 | 0.407 | 1.250 | 0.956-1.633 | 0.102 |
| **2. Psychological factors** |  |  |  |  |  |  |  |  |  |
| PHQ-9 (per SD) | 3.751 | 2.685-5.241 | **<0.001** | 3.205 | 2.027-5.066 | **<0.001** | 5.213 | 3.549-7.657 | **<0.001** |
| GAD-7 (per SD) | 2.453 | 1.806-3.331 | **<0.001** | 1.925 | 1.231-3.008 | **0.004** | 3.055 | 2.161-4.319 | **<0.001** |
| CFS (per SD) | 1.986 | 1.587-2.484 | **<0.001** | 2.244 | 1.527-3.299 | **<0.001** | 3.560 | 2.561-4.949 | **<0.001** |
| **3. Polysomnographic factors** | | | | | | | | | |
| **3.1** **Sleep stages and arousals** | | | | | | | | | |
| Total sleep time (per SD) | 0.833 | 0.674-1.029 | 0.090 | 1.239 | 0.846-1.814 | 0.271 | 0.987 | 0.748-1.303 | 0.927 |
| Sleep efficiency (per SD) | 0.818 | 0.655-1.022 | 0.078 | 1.522 | 0.947-2.446 | 0.083 | 0.858 | 0.642-1.148 | 0.303 |
| Sleep latency (per SD) | 1.558 | 1.149-2.113 | **0.004** | 0.147 | 0.037-0.594 | **0.007** | 1.252 | 0.854-1.837 | 0.249 |
| REM sleep latency (per SD) | 1.030 | 0.843-1.259 | 0.770 | 0.686 | 0.451-1.041 | 0.077 | 0.883 | 0.671-1.161 | 0.372 |
| N1% (per SD) | 0.722 | 0.580-0.898 | **0.004** | 0.901 | 0.627-1.294 | 0.571 | 0.878 | 0.666-1.156 | 0.353 |
| N2% (per SD) | 1.378 | 1.119-1.699 | **0.003** | 1.055 | 0.741-1.503 | 0.767 | 1.011 | 0.772-1.322 | 0.939 |
| N3% (per SD) | 1.153 | 0.932-1.427 | 0.191 | 0.863 | 0.581-1.282 | 0.467 | 0.966 | 0.722-1.292 | 0.813 |
| REM% (per SD) | 0.993 | 0.804-1.227 | 0.948 | 1.455 | 1.021-2.074 | **0.038** | 1.453 | 1.101-1.917 | **0.008** |
| Total arousal index (per SD) | 0.857 | 0.692-1.062 | 0.159 | 0.985 | 0.711-1.365 | 0.929 | 0.880 | 0.673-1.150 | 0.349 |
| Respiration-related arousal index (per SD) | 0.823 | 0.659-1.028 | 0.086 | 1.079 | 0.793-1.466 | 0.629 | 0.902 | 0.693-1.175 | 0.445 |
| Respiration-related arousal index: NREM (per SD) | 0.808 | 0.646-1.010 | 0.061 | 1.078 | 0.795-1.461 | 0.630 | 0.899 | 0.692-1.170 | 0.429 |
| Respiration-related arousal index: REM (per SD) | 0.912 | 0.739-1.125 | 0.389 | 1.010 | 0.719-1.420 | 0.953 | 0.870 | 0.659-1.149 | 0.326 |
| Spontaneous arousal index (per SD) | 1.021 | 0.841-1.240 | 0.834 | 0.609 | 0.378-0.980 | **0.041** | 0.805 | 0.594-1.091 | 0.162 |
| Spontaneous arousal index: NREM (per SD) | 1.022 | 0.841-1.241 | 0.828 | 0.625 | 0.390-1.000 | 0.050 | 0.789 | 0.580-1.074 | 0.132 |
| **3.2 Parameters of OSA severity and hypoxemia** | | | | | | | | | |
| AHI (per SD) | 0.783 | 0.625-0.981 | **0.033** | 1.231 | 0.872-1.737 | 0.238 | 0.888 | 0.674-1.169 | 0.396 |
| ODI (per SD) | 0.844 | 0.671-1.061 | 0.147 | 1.286 | 0.904-1.830 | 0.162 | 0.978 | 0.737-1.296 | 0.874 |
| AI (per SD) | 0.842 | 0.671-1.056 | 0.136 | 1.437 | 1.043-1.979 | **0.027** | 1.073 | 0.825-1.395 | 0.599 |
| HI (per SD) | 0.891 | 0.728-1.090 | 0.263 | 0.698 | 0.482-1.011 | 0.057 | 0.717 | 0.544-0.947 | **0.019** |
| AHI_NREM_ (per SD) | 0.806 | 0.649-1.002 | 0.052 | 1.196 | 0.854-1.674 | 0.297 | 0.932 | 0.717-1.211 | 0.598 |
| AHI_REM_ (per SD) | 1.101 | 0.893-1.358 | 0.366 | 1.599 | 1.123-2.276 | **0.009** | 1.276 | 0.974-1.671 | 0.077 |
| AHI_nonsupine_ (per SD) | 0.869 | 0.692-1.092 | 0.228 | 1.509 | 1.083-2.102 | **0.015** | 1.064 | 0.811-1.396 | 0.654 |
| AHI_supine_ (per SD) | 0.842 | 0.674-1.051 | 0.128 | 1.094 | 0.769-1.557 | 0.618 | 0.931 | 0.706-1.227 | 0.611 |
| AI_NREM_ (per SD) | 0.794 | 0.619-1.017 | 0.068 | 1.338 | 0.966-1.852 | 0.080 | 1.004 | 0.764-1.319 | 0.979 |
| TST90 (per SD) | 0.858 | 0.678-1.085 | 0.200 | 1.127 | 0.833-1.524 | 0.438 | 1.066 | 0.835-1.362 | 0.607 |
| Mean oxygen desaturation (per SD) | 0.858 | 0.626-1.175 | 0.339 | 0.811 | 0.517-1.274 | 0.364 | 1.082 | 0.785-1.492 | 0.630 |
| Lowest oxygen saturation during sleep (per SD) | 1.251 | 0.999-1.567 | 0.051 | 0.909 | 0.646-1.279 | 0.584 | 0.889 | 0.684-1.156 | 0.380 |
| Mean oxygen saturation during sleep (per SD) | 1.113 | 0.883-1.404 | 0.365 | 0.846 | 0.609-1.174 | 0.317 | 0.952 | 0.729-1.244 | 0.720 |
| Mean duration of apnea (per SD) | 0.984 | 0.798-1.214 | 0.881 | 0.832 | 0.577-1.200 | 0.324 | 0.929 | 0.695-1.243 | 0.620 |
| Longest duration of hypopnea (per SD) | 0.938 | 0.768-1.147 | 0.534 | 0.589 | 0.388-0.896 | **0.013** | 0.918 | 0.694-1.214 | 0.547 |
| Mean duration of hypopnea (per SD) | 0.962 | 0.773-1.196 | 0.725 | 0.767 | 0.508-1.159 | 0.208 | 0.948 | 0.701-1.283 | 0.730 |
| **3.3 Limb movements and heart rate** | | | | | | | | | |
| Highest HR during sleep (per SD) | 0.832 | 0.669-1.035 | 0.098 | 1.142 | 0.785-1.662 | 0.487 | 0.911 | 0.682-1.219 | 0.531 |

The demographic model included age, sex, and BMI simultaneously. Each psychological or polysomnographic variable was entered separately into a multinomial logistic regression model adjusted for age, sex, and BMI. The low symptom burden group was defined as the reference group.

All continuous variables were Z-score standardized, and ORs for continuous variables are expressed per 1-SD increase.

P values were not adjusted for multiple comparisons. Nominally statistically significant results (two-sided P<0.05) are shown in bold and should be interpreted together with the effect estimates, 95% CIs, and sensitivity analyses.

OSA, obstructive sleep apnea; EDS, excessive daytime sleepiness; OR, odds ratio; CI, confidence interval; SD, standard deviation; BMI, body mass index; GAD-7, Generalized Anxiety Disorder-7; PHQ-9, Patient Health Questionnaire-9; CFS, Chalder Fatigue Scale; REM, rapid eye movement; N1%, percentage of N1 relative to total sleep time; N2%, percentage of N2 relative to total sleep time; N3%, percentage of N3 relative to total sleep time; REM%, percentage of REM relative to total sleep time; NREM, non-rapid eye movement; AHI, apnea-hypopnea index; ODI, oxygen desaturation index; AI, apnea index; HI, hypopnea index; AHI_NREM_, AHI during NREM sleep; AHI_REM_, AHI during REM sleep; AHI_nonsupine_, AHI in non-supine position; AHI_supine_, AHI in supine position; AI_NREM_, apnea index during NREM sleep; HI_NREM_, hypopnea index during NREM sleep; TST90, percentage of total sleep time with SpO2 < 90%; HR, heart rate.

**Supplementary Table 4** Multinomial logistic regression analyses of factors associated with OSA symptom-groups among patients without any coexisting sleep disorder.

| **Variables** | **Poor sleep quality only vs Low symptom burden** | | | **EDS only vs Low symptom burden** | | | **Combined vs Low symptom burden** | | |
| --- | --- | --- | --- | --- | --- | --- | --- | --- | --- |
|  | **OR** | **95% CI** | ***P*** | **OR** | **95% CI** | ***P*** | **OR** | **95% CI** | ***P*** |
| **1. Demographic factors** | | | | | | | | | |
| Age (per SD) | 1.129 | 0.907–1.405 | 0.278 | 0.752 | 0.503–1.126 | 0.167 | 1.201 | 0.892–1.617 | 0.227 |
| Sex, male | 0.510 | 0.308–0.845 | **0.009** | 0.688 | 0.264–1.795 | 0.445 | 0.944 | 0.465–1.918 | 0.874 |
| BMI (per SD) | 0.674 | 0.543–0.836 | **<0.001** | 1.238 | 0.884–1.733 | 0.215 | 1.130 | 0.863–1.481 | 0.374 |
| **2. Psychological factors** | | | | | | | | | |
| GAD-7 (per SD) | 2.275 | 1.681–3.079 | **<0.001** | 1.506 | 0.905–2.506 | 0.115 | 2.730 | 1.928–3.865 | **<0.001** |
| PHQ-9 (per SD) | 3.454 | 2.460–4.849 | **<0.001** | 3.339 | 2.082–5.353 | **<0.001** | 4.310 | 2.925–6.349 | **<0.001** |
| **3. Polysomnographic parameters** | | | | | | | | | |
| **3.1 Sleep stage and arousals** | | | | | | | | | |
| Total sleep time (per SD) | 0.778 | 0.626–0.966 | **0.023** | 1.202 | 0.796–1.816 | 0.382 | 0.959 | 0.719–1.278 | 0.773 |
| Sleep efficiency (per SD) | 0.814 | 0.649–1.023 | 0.077 | 1.220 | 0.755–1.970 | 0.417 | 0.941 | 0.693–1.278 | 0.696 |
| Sleep onset latency (per SD) | 1.343 | 1.026–1.760 | **0.032** | 0.099 | 0.021–0.471 | **0.004** | 1.063 | 0.720–1.568 | 0.758 |
| REM sleep latency (per SD) | 1.043 | 0.848–1.283 | 0.690 | 0.824 | 0.540–1.257 | 0.368 | 0.899 | 0.674–1.201 | 0.472 |
| N1% (per SD) | 0.685 | 0.542–0.866 | **0.002** | 1.155 | 0.795–1.679 | 0.449 | 0.816 | 0.609–1.092 | 0.171 |
| N2% (per SD) | 1.421 | 1.143–1.767 | **0.002** | 0.850 | 0.583–1.241 | 0.400 | 1.040 | 0.789–1.372 | 0.781 |
| N3% (per SD) | 1.211 | 0.966–1.519 | 0.097 | 0.806 | 0.521–1.249 | 0.335 | 1.126 | 0.835–1.518 | 0.435 |
| REM% (per SD) | 0.936 | 0.748–1.173 | 0.567 | 1.263 | 0.826–1.929 | 0.281 | 1.339 | 0.985–1.821 | 0.062 |
| Total arousal index (per SD) | 0.704 | 0.551–0.901 | **0.005** | 1.203 | 0.885–1.636 | 0.238 | 0.829 | 0.620–1.108 | 0.205 |
| Respiration-related arousal index (per SD) | 0.699 | 0.550–0.889 | **0.003** | 1.226 | 0.909–1.653 | 0.183 | 0.854 | 0.648–1.125 | 0.262 |
| Respiration-related arousal index: NREM (per SD) | 0.687 | 0.541–0.873 | **0.002** | 1.218 | 0.906–1.637 | 0.192 | 0.849 | 0.645–1.117 | 0.242 |
| Respiration-related arousal index: REM (per SD) | 0.839 | 0.663–1.062 | 0.144 | 1.220 | 0.857–1.737 | 0.269 | 0.885 | 0.662–1.184 | 0.411 |
| LM-related arousal index (per SD) | 0.897 | 0.709–1.135 | 0.367 | 1.226 | 0.848–1.774 | 0.278 | 1.200 | 0.912–1.580 | 0.193 |
| LM-related arousal index: REM (per SD) | 1.352 | 1.009–1.811 | **0.043** | 1.303 | 0.859–1.976 | 0.214 | 1.374 | 0.988–1.910 | 0.059 |
| PLM-related arousal index (per SD) | 0.866 | 0.547–1.370 | 0.539 | 1.339 | 0.681–2.633 | 0.398 | 1.640 | 1.064–2.528 | **0.025** |
| Spontaneous arousal index (per SD) | 1.045 | 0.817–1.337 | 0.725 | 0.818 | 0.495–1.351 | 0.432 | 0.774 | 0.540–1.110 | 0.164 |
| Spontaneous arousal index: NREM (per SD) | 1.033 | 0.810–1.319 | 0.792 | 0.855 | 0.525–1.395 | 0.531 | 0.763 | 0.533–1.093 | 0.140 |
| **3.2 Parameters of OSA severity and hypoxemia** | | | | | | | | | |
| AHI (per SD) | 0.721 | 0.571–0.912 | **0.006** | 1.335 | 0.911–1.956 | 0.138 | 0.820 | 0.610–1.101 | 0.187 |
| ODI (per SD) | 0.763 | 0.605–0.963 | **0.023** | 1.264 | 0.874–1.830 | 0.214 | 0.866 | 0.649–1.157 | 0.331 |
| AI (per SD) | 0.768 | 0.615–0.958 | **0.019** | 1.385 | 1.010–1.899 | **0.043** | 0.989 | 0.768–1.273 | 0.932 |
| HI (per SD) | 0.889 | 0.727–1.088 | 0.255 | 0.854 | 0.596–1.222 | 0.388 | 0.732 | 0.553–0.971 | **0.030** |
| AHI_NREM_ (per SD) | 0.716 | 0.565–0.907 | **0.006** | 1.291 | 0.888–1.878 | 0.181 | 0.817 | 0.609–1.098 | 0.180 |
| AHI_REM_ (per SD) | 1.127 | 0.912–1.391 | 0.268 | 1.662 | 1.144–2.413 | **0.008** | 1.196 | 0.911–1.571 | 0.198 |
| AHI_nonsupine_ (per SD) | 0.783 | 0.626–0.980 | **0.032** | 1.531 | 1.101–2.130 | **0.011** | 1.045 | 0.805–1.356 | 0.739 |
| AHI_supine_ (per SD) | 0.749 | 0.591–0.949 | **0.017** | 1.174 | 0.792–1.740 | 0.424 | 0.876 | 0.647–1.186 | 0.392 |
| AI_NREM_ (per SD) | 1.003 | 0.810–1.242 | 0.979 | 1.380 | 0.979–1.944 | 0.066 | 1.027 | 0.785–1.344 | 0.845 |
| HI_NREM_ (per SD) | 0.857 | 0.700–1.051 | 0.138 | 0.860 | 0.602–1.229 | 0.408 | 0.739 | 0.557–0.980 | **0.036** |
| TST90 (per SD) | 0.834 | 0.668–1.042 | 0.109 | 1.124 | 0.854–1.481 | 0.405 | 0.945 | 0.743–1.202 | 0.645 |
| Mean oxygen desaturation (per SD) | 0.890 | 0.668–1.186 | 0.428 | 0.863 | 0.566–1.316 | 0.493 | 1.062 | 0.780–1.445 | 0.703 |
| Lowest oxygen saturation during sleep (per SD) | 1.337 | 1.071–1.668 | **0.010** | 0.945 | 0.668–1.338 | 0.752 | 1.023 | 0.785–1.333 | 0.867 |
| Mean oxygen saturation during sleep (per SD) | 1.225 | 0.969–1.547 | 0.089 | 0.864 | 0.622–1.200 | 0.383 | 1.049 | 0.798–1.380 | 0.730 |
| Longest duration of apnea (per SD) | 0.786 | 0.630–0.981 | **0.033** | 1.004 | 0.663–1.521 | 0.985 | 1.030 | 0.773–1.372 | 0.839 |
| Mean duration of apnea (per SD) | 0.852 | 0.683–1.064 | 0.158 | 0.821 | 0.543–1.244 | 0.353 | 0.989 | 0.725–1.350 | 0.945 |
| Longest duration of hypopnea (per SD) | 0.931 | 0.755–1.147 | 0.499 | 0.699 | 0.452–1.080 | 0.107 | 0.917 | 0.692–1.215 | 0.545 |
| Mean duration of hypopnea (per SD) | 0.933 | 0.752–1.159 | 0.532 | 0.843 | 0.550–1.292 | 0.432 | 0.909 | 0.677–1.220 | 0.525 |
| **3.3 Limb movements and heart rate** | | | | | | | | | |
| PLM index of sleep (per SD) | 0.990 | 0.754–1.299 | 0.940 | 0.867 | 0.475–1.583 | 0.643 | 1.285 | 0.944–1.749 | 0.111 |
| PLM index: NREM (per SD) | 1.008 | 0.759–1.338 | 0.957 | 0.894 | 0.483–1.653 | 0.720 | 1.319 | 0.957–1.819 | 0.090 |
| PLM index: REM (per SD) | 0.617 | 0.392–0.974 | **0.038** | 0.861 | 0.456–1.627 | 0.646 | 0.953 | 0.721–1.259 | 0.733 |
| Highest HR during sleep (per SD) | 0.911 | 0.728–1.140 | 0.414 | 1.441 | 0.957–2.170 | 0.080 | 0.942 | 0.699–1.269 | 0.693 |
| Arrhythmia | 0.787 | 0.347–1.782 | 0.566 | 2.685 | 0.914–7.885 | 0.072 | 1.357 | 0.526–3.497 | 0.528 |

The demographic model included age, sex, and BMI simultaneously. Each psychological or polysomnographic variable was entered separately into a multinomial logistic regression model adjusted for age, sex, and BMI. The low symptom burden group was defined as the reference group.

All continuous variables were Z-score standardized, and ORs for continuous variables are expressed per 1-SD increase.

P values were not adjusted for multiple comparisons. Nominally statistically significant results (two-sided P<0.05) are shown in bold and should be interpreted together with the effect estimates, 95% CIs, and sensitivity analyses.

OSA, obstructive sleep apnea; EDS, excessive daytime sleepiness; OR, odds ratio; CI, confidence interval; SD, standard deviation; BMI, body mass index; GAD-7, Generalized Anxiety Disorder-7; PHQ-9, Patient Health Questionnaire-9; REM, rapid eye movement; N1%, percentage of N1 relative to total sleep time; N2%, percentage of N2 relative to total sleep time; N3%, percentage of N3 relative to total sleep time; REM%, percentage of REM relative to total sleep time; NREM, non-rapid eye movement; LM, limb movement; PLM, periodic limb movement; AHI, apnea-hypopnea index; ODI, oxygen desaturation index; AI, apnea index; HI, hypopnea index; AHI_NREM_, AHI during NREM sleep; AHI_REM_, AHI during REM sleep; AHI_nonsupine_, AHI in non-supine position; AHI_supine_, AHI in supine position; AI_NREM_, apnea index during NREM sleep; HI_NREM_, hypopnea index during NREM sleep; TST90, percentage of total sleep time with SpO2 < 90%; HR, heart rate.

**Supplementary Table 5** Multinomial logistic regression analyses of polysomnographic correlates with additional adjustment for AHI.

| **Polysomnographic parameters** | **Poor sleep quality only vs Low symptom burden** | | | **EDS only vs Low symptom burden** | | | **Combined vs Low symptom burden** | | |
| --- | --- | --- | --- | --- | --- | --- | --- | --- | --- |
|  | **OR** | **95% CI** | **P** | **OR** | **95% CI** | **P** | **OR** | **95% CI** | **P** |
| **1 Sleep stage and arousals** | | | | | | | | | |
| Total sleep time (per SD) | 0.803 | 0.670–0.961 | **0.017** | 1.290 | 0.922–1.807 | 0.138 | 1.017 | 0.798–1.297 | 0.891 |
| Sleep efficiency (per SD) | 0.811 | 0.671–0.980 | **0.030** | 1.383 | 0.926–2.066 | 0.113 | 0.971 | 0.749–1.260 | 0.827 |
| Sleep onset latency (per SD) | 1.382 | 1.102–1.734 | **0.005** | 0.127 | 0.037–0.442 | **0.001** | 1.098 | 0.803–1.502 | 0.556 |
| REM sleep latency (per SD) | 1.094 | 0.919–1.302 | 0.313 | 0.671 | 0.463–0.973 | **0.035** | 0.885 | 0.693–1.131 | 0.329 |
| N1% (per SD) | 0.802 | 0.623–1.031 | 0.085 | 0.907 | 0.598–1.377 | 0.648 | 1.018 | 0.740–1.400 | 0.915 |
| N2% (per SD) | 1.242 | 1.026–1.505 | **0.027** | 1.040 | 0.742–1.457 | 0.821 | 0.938 | 0.728–1.209 | 0.621 |
| N3% (per SD) | 1.000 | 0.823–1.216 | 0.998 | 0.789 | 0.544–1.145 | 0.212 | 0.873 | 0.665–1.146 | 0.328 |
| REM% (per SD) | 0.943 | 0.783–1.135 | 0.534 | 1.443 | 1.054–1.975 | **0.022** | 1.295 | 1.012–1.656 | **0.040** |
| Total arousal index (per SD) | 1.062 | 0.825–1.366 | 0.643 | 0.853 | 0.543–1.340 | 0.490 | 0.992 | 0.712–1.382 | 0.964 |
| Respiration-related arousal index (per SD) | 1.011 | 0.715–1.432 | 0.949 | 0.882 | 0.515–1.510 | 0.646 | 0.975 | 0.636–1.497 | 0.909 |
| Respiration-related arousal index: NREM (per SD) | 0.958 | 0.679–1.352 | 0.809 | 0.884 | 0.524–1.492 | 0.645 | 0.971 | 0.641–1.471 | 0.890 |
| Respiration-related arousal index: REM (per SD) | 1.081 | 0.852–1.371 | 0.521 | 0.876 | 0.577–1.329 | 0.533 | 0.875 | 0.632–1.211 | 0.421 |
| LM-related arousal index (per SD) | 0.945 | 0.792–1.127 | 0.527 | 1.164 | 0.867–1.565 | 0.312 | 1.212 | 0.977–1.502 | 0.080 |
| LM-related arousal index: REM (per SD) | 1.211 | 0.982–1.495 | 0.074 | 1.310 | 0.984–1.745 | 0.064 | 1.244 | 0.971–1.594 | 0.085 |
| PLM-related arousal index (per SD) | 1.159 | 0.926–1.450 | 0.197 | 1.076 | 0.671–1.726 | 0.762 | 1.322 | 1.029–1.699 | **0.029** |
| Spontaneous arousal index (per SD) | 1.040 | 0.877–1.233 | 0.655 | 0.823 | 0.563–1.203 | 0.315 | 0.894 | 0.689–1.159 | 0.397 |
| Spontaneous arousal index: NREM (per SD) | 1.034 | 0.872–1.227 | 0.701 | 0.856 | 0.592–1.237 | 0.407 | 0.890 | 0.687–1.154 | 0.380 |
| **2 Parameters of OSA severity and hypoxemia** | | | | | | | | | |
| ODI (per SD) | 1.304 | 0.840–2.027 | 0.237 | 1.337 | 0.601–2.975 | 0.477 | 1.610 | 0.862–3.009 | 0.135 |
| AI (per SD) | 1.120 | 0.801–1.567 | 0.508 | 1.960 | 1.138–3.374 | **0.015** | 1.651 | 1.076–2.534 | **0.022** |
| HI (per SD) | 0.933 | 0.760–1.145 | 0.508 | 0.664 | 0.477–0.924 | **0.015** | 0.737 | 0.568–0.956 | **0.022** |
| AHI_NREM_ (per SD) | 0.924 | 0.675–1.264 | 0.619 | 1.009 | 0.605–1.682 | 0.973 | 1.111 | 0.716–1.724 | 0.640 |
| AHI_REM_ (per SD) | 1.186 | 0.971–1.448 | 0.095 | 1.391 | 0.996–1.943 | 0.052 | 1.258 | 0.971–1.630 | 0.082 |
| AHI_nonsupine_ (per SD) | 1.095 | 0.837–1.432 | 0.507 | 1.648 | 1.082–2.510 | **0.020** | 1.345 | 0.958–1.888 | 0.087 |
| AHI_supine_ (per SD) | 1.161 | 0.792–1.702 | 0.445 | 0.706 | 0.364–1.367 | 0.302 | 1.106 | 0.676–1.812 | 0.688 |
| AI_NREM_ (per SD) | 0.963 | 0.685–1.354 | 0.830 | 1.660 | 1.004–2.744 | **0.048** | 1.414 | 0.942–2.123 | 0.094 |
| HI_NREM_ (per SD) | 0.950 | 0.761–1.185 | 0.649 | 0.656 | 0.460–0.936 | **0.020** | 0.749 | 0.568–0.989 | **0.041** |
| TST90 (per SD) | 1.042 | 0.834–1.302 | 0.718 | 1.007 | 0.712–1.424 | 0.967 | 1.191 | 0.927–1.531 | 0.172 |
| Mean oxygen desaturation (per SD) | 0.977 | 0.729–1.308 | 0.873 | 0.626 | 0.414–0.946 | **0.026** | 1.238 | 0.877–1.746 | 0.224 |
| Lowest oxygen saturation during sleep (per SD) | 1.116 | 0.886–1.405 | 0.353 | 1.126 | 0.764–1.659 | 0.548 | 0.814 | 0.613–1.080 | 0.154 |
| Mean oxygen saturation during sleep (per SD) | 0.925 | 0.726–1.177 | 0.526 | 0.994 | 0.672–1.470 | 0.977 | 0.843 | 0.623–1.140 | 0.267 |
| Longest duration of apnea (per SD) | 0.944 | 0.764–1.166 | 0.592 | 0.731 | 0.482–1.108 | 0.140 | 1.091 | 0.823–1.446 | 0.545 |
| Mean duration of apnea (per SD) | 0.982 | 0.811–1.189 | 0.851 | 0.843 | 0.584–1.218 | 0.363 | 0.996 | 0.761–1.303 | 0.976 |
| Longest duration of hypopnea (per SD) | 0.904 | 0.764–1.070 | 0.241 | 0.604 | 0.418–0.872 | **0.007** | 0.849 | 0.667–1.081 | 0.184 |
| Mean duration of hypopnea (per SD) | 0.924 | 0.769–1.111 | 0.403 | 0.716 | 0.491–1.044 | 0.083 | 0.900 | 0.693–1.168 | 0.428 |
| **3 Limb movements and heart rate** | | | | | | | | | |
| PLM index of sleep (per SD) | 1.033 | 0.863–1.236 | 0.726 | 0.963 | 0.645–1.439 | 0.855 | 1.254 | 1.004–1.568 | **0.046** |
| PLM index: NREM (per SD) | 1.061 | 0.883–1.274 | 0.527 | 0.943 | 0.621–1.434 | 0.785 | 1.288 | 1.028–1.614 | **0.028** |
| PLM index: REM (per SD) | 0.920 | 0.781–1.083 | 0.316 | 1.056 | 0.806–1.382 | 0.694 | 0.973 | 0.779–1.216 | 0.813 |
| Highest HR during sleep (per SD) | 0.861 | 0.711–1.042 | 0.124 | 0.968 | 0.686–1.368 | 0.855 | 1.020 | 0.789–1.317 | 0.881 |
| Arrhythmia | 0.673 | 0.345–1.313 | 0.245 | 1.880 | 0.750–4.712 | 0.178 | 0.890 | 0.374–2.118 | 0.793 |

Each polysomnographic variable was entered separately into a multinomial logistic regression model adjusted for age, sex, BMI, and AHI. The low symptom burden group was defined as the reference group.

All continuous variables were Z-score standardized, and ORs for continuous variables are expressed per 1-SD increase.

P values were not adjusted for multiple comparisons. Nominally statistically significant results (two-sided P<0.05) are shown in bold and should be interpreted together with the effect estimates, 95% CIs, and sensitivity analyses.

AHI, apnea-hypopnea index; EDS, excessive daytime sleepiness; OR, odds ratio; CI, confidence interval; SD, standard deviation; REM, rapid eye movement; N1%, percentage of N1 relative to total sleep time; N2%, percentage of N2 relative to total sleep time; N3%, percentage of N3 relative to total sleep time; REM%, percentage of REM relative to total sleep time; NREM, non-rapid eye movement; LM, limb movement; PLM, periodic limb movement; ODI, oxygen desaturation index; AI, apnea index; HI, hypopnea index; AHI_NREM_, AHI during NREM sleep; AHI_REM_, AHI during REM sleep; AHI_nonsupine_, AHI in non-supine position; AHI_supine_, AHI in supine position; AI_NREM_, apnea index during NREM sleep; HI_NREM_, hypopnea index during NREM sleep; TST90, percentage of total sleep time with SpO2 < 90%; HR, heart rate.
